# Supplementary material for: European Autism GEnomics Registry (EAGER): protocol for a multicentre cohort study and registry
Source: BMJ Open. 2024 Jun 4;14(6):e080746. doi: 10.1136/bmjopen-2023-080746 (PMC11163653; doi:10.1136/bmjopen-2023-080746)
Supplement: Author's manuscript [file bmjopen-2023-080746.draft_revisions.pdf]

BMJ Open is committed to open peer review. As part of this commitment we make the peer review history of every article we publish publicly available.

When an article is published we post the peer reviewers' comments and the authors' responses online. We also post the versions of the paper that were used during peer review. These are the versions that the peer review comments apply to.

The versions of the paper that follow are the versions that were submitted during the peer review process. They are not the versions of record or the final published versions. They should not be cited or distributed as the published version of this manuscript.

BMJ Open is an open access journal and the full, final, typeset and author-corrected version of record of the manuscript is available on our site with no access controls, subscription charges or pay-per-view fees (http://bmjopen.bmj.com).

If you have any questions on BMJ Open's open peer review process please email info.bmjopen@bmj.com

BMJ Open

European Autism GEnomics Registry (EAGER): Protocol for a multicentre cohort study and registry

Journal:	*BMJ Open*
Manuscript ID	bmjopen-2023-080746
Article Type:	Protocol
Date Submitted by the Author:	09-Oct-2023
Complete List of Authors:	Bloomfield, Madeleine; King's College London, Psychology, Institute of Psychiatry, Psychology & Neuroscience<br>Lautarescu, Alexandra; King's College London, Psychology, Institute of Psychiatry, Psychology & Neuroscience<br>Heraty, Síofra; Birkbeck University of London, Department of Psychological Sciences<br>Douglas, Sarah; Cambridge University, AIMS-2-TRIALS A-Reps<br>Violland, Pierre; Cambridge University, AIMS-2-TRIALS A-Reps<br>Plas, Roderik; Cambridge University, AIMS-2-TRIALS A-Reps<br>Ghosh, Anjuli; Cambridge University, AIMS-2-TRIALS A-Reps<br>Van den Bosch, Katrien; Cambridge University, AIMS-2-TRIALS A-Reps<br>Eaton, Eliza; Cambridge University, Autism Research Centre<br>Absound, Michael; Guy's and St Thomas' Hospitals NHS Trust, Department of Children's Neurosciences, Evelina London Children's Hospital; King's College London, Department of Women and Children's Health, Faculty of Life Sciences and Medicine, School of Life Course Sciences<br>Battini, Roberta ; IRCCS Fondazione Stella Maris, Department of Developmental Neuroscience<br>Blázquez Hinojosa, Ana ; Hospital Clinic de Barcelona, Department of Child and Adolescent Psychiatry and Psychology, Institute of Neurosciences<br>Bolshakova, Nadia; Trinity College Dublin, Department of Psychiatry<br>Bölte, Sven; Karolinska Institutet, Center of Neurodevelopmental Disorders (KIND), Department of Women's and Children's Health, Centre for Psychiatry Research; Region Stockholm, Child and Adolescent Psychiatry, Stockholm Health Care Services<br>Bonanni, Paolo; IRCCS Eugenio Medea, Epilepsy Unit<br>Borg, Jacqueline ; Karolinska Institutet, Centre for Psychiatry Research and Centre for Cognitive and Computational Neuropsychiatry (CCNP), Department of Clinical Neuroscience; Sahlgrenska University Hospital, Psykiatri Affektiva<br>Calderoni, Sara; IRCCS Fondazione Stella Maris, Department of Developmental Neuroscience; University of Pisa, Department of Clinical and Experimental Medicine<br>Calvo Escalona, Rosa; Hospital Clinic de Barcelona, Department of Child and Adolescent Psychiatry and Psychology, Institute of Neurosciences; Institut d'Investigacions Biomèdiques August Pi i Sunyer<br>Castelo-Branco, Miguel; University of Coimbra, CIBIT, ICNAS, Faculty of Medicine

Castro-Fornieles, Josefina; Hospital Clinic de Barcelona, Department of Child and Adolescent Psychiatry and Psychology, 2021SGR01319; Institut d'Investigacions Biomèdiques August Pi i Sunyer
Caro, Pilar; University Hospital Heidelberg, Institute of Human Genetics
Danieli, Alberto; IRCCS Eugenio Medea, Epilepsy Unit
Delorme, Richard; Universite Paris Cite, Child and Adolescent Psychiatry Department, Robert Debre Hospital, APHP
Elia, Maurizio; Oasi Research Institute-IRCCS, Unit of Neurology and Clinical Neurophysiopathology
Hempel, Maja; Heidelberg University, Institute of Human Genetics
Madeira, Nuno; Centro Hospitalar e Universitario de Coimbra EPE, Psychiatry Department; University of Coimbra, Coimbra Institute for Biomedical Imaging and Translational Research
McAlonan, Grainne; King's College London, Department of Forensic and Neurodevelopmental Sciences, Institute of Psychiatry, Psychology, & Neuroscience; South London and Maudsley NHS Foundation Trust, Behavioural and Developmental Clinical Academic Group
Milone, Roberta; IRCCS Fondazione Stella Maris, Department of Developmental Neuroscience
Molloy, Ciara J.; Trinity College Dublin, Department of Psychiatry
Mouga, Susana; University of Coimbra, Coimbra Institute for Biomedical Imaging and Translational Research; University of Coimbra, Institute of Nuclear Sciences Applied to Health
Montiel, Virginia; Institut de Recerca Sant Joan de Deu, Pediatric Neurology Department, Hospital Sant Joan de Déu
Pina Rodrigues, Ana; University of Coimbra, Coimbra Institute for Biomedical Imaging and Translational Research, Institute of Nuclear Sciences Applied to Health
Schaaf, Christian P.; University Hospital Heidelberg, Institute of Human Genetics
Serrano, Mercedes; Institut de Recerca Sant Joan de Deu, Pediatric Neurology Department, Hospital Sant Joan de Déu
Tammimies, Kristiina; Karolinska Institutet, Center of Neurodevelopmental Disorders (KIND), Department of Women’s and Children’s Health, Centre for Psychiatry Research; Region Stockholm, Astrid Lindgren Children's Hospital, Karolinska University Hospital
Tye, Charlotte; King's College London, Psychology, Institute of Psychiatry, Psychology & Neuroscience
Vigevano, Federico; Bambino Gesù Children\'s Hospital, IRCCS, Neurological Sciences and Rehabilitation Medicine Scientific Area
Oliveira, Guiomar; University of Coimbra, University Clinic of Pediatrics, Faculty of Medicine; Centro Hospitalar e Universitario de Coimbra EPE, Child Developmental Center and Research and Clinical Training Center, Pediatric Hospital
Mazzone, Beatrice; IRCCS Fondazione Stella Maris, Department of Developmental Neuroscience
O’Neill, Cara ; Cure Sanfilippo Foundation
Romero, Verena; Dup15q Alliance
Tillmann, Julian; Roche Innovation Center Basel, Roche Pharma Research and Early Development
Oakley, Bethany; King's College London, Department of Forensic and Neurodevelopmental Sciences, Psychology, Institute of Psychiatry, Psychology & Neuroscience
Murphy, Declan G. M.; King's College London, Department of Forensic and Neurodevelopmental Sciences, Psychology, Institute of Psychiatry, Psychology & Neuroscience; South London and Maudsley NHS Foundation Trust
Gallagher , Louise ; SickKids Department of Psychiatry; CAMH, Child and Youth Division Centre for Addiction and Mental Health
Bourgeron, Thomas; Institut Pasteur, Génétique Humaine et Fonctions Cognitives

	Chatham, Christopher; F Hoffmann-La Roche Ltd<br>Charman, Tony; King's College London, Department of Psychology, Institute of Psychiatry, Psychology & Neuroscience
Keywords:	GENETICS, REGISTRIES, MENTAL HEALTH

SCHOLARONE™
Manuscripts

European Autism GEnomics Registry (EAGER):

Protocol for a multicentre cohort study and registry

Authors:

Bloomfield, M.[1*], Lautarescu, A.[1*], Heraty, S.[2], Douglas, S.[3], Violland, P.[3], Plas, R.[3], Ghosh, A.[3], Van den Bosch, K.[3], Eaton, E.[4], Absoud, M.[5,6], Battini, R.[7,8], Blázquez Hinojosa, A.[9], Bolshakova, N.[10], Bolte, S.[11,12,13], Bonanni, P.[14], Borg, J.[15,16], Calderoni, S.[7,8], Calvo Escalona, R.[9,17,18,19], Castelo-Branco, M.[20], Castro-Fornieles, J.[9,17,18,19], Caro P.[21], Danieli, A.[14], Delorme, R.[22], Elia, M.[23], Hempel, M.[21], Madeira, N.[24,25,26], McAlonan, G.[27,28], Milone, R.[7], Molloy, C. J.[10], Mouga, S.[29,30], Montiel, V.[31], Pina Rodrigues, A.[29,30], Schaaf, C. P.[21], Serrano, M.[31], Tammimies, K.[11,32], Tye, C.[1], Vigevano, F.[33], Oliveira, G.[34,35], Mazzone, B.[7], O'Neill, C.[36], Romero, V.[37], Tillmann, J.[38], Oakley, B.[27], Murphy, D.[27,39,40], Gallagher, L.[10,41,42,43], Bourgeron, T.[44], Chatham, C.[45], Charman, T.[1]

* Indicates equal contribution

Affiliations:

1 - Department of Psychology, Institute of Psychiatry, Psychology & Neuroscience, King's College London, London, United Kingdom

2 - Department of Psychological Sciences, University of London, Birkbeck, London, United Kingdom

3 - AIMS-2-Trials A-Reps, University of Cambridge, Cambridge, United Kingdom

4 - Autism Research Centre, University of Cambridge, Cambridge, United Kingdom

For peer review only

5 - Department of Children's Neurosciences, Evelina London Children's Hospital, Guy's and St Thomas' NHS Foundation Trust, London, United Kingdom

6 - Department of Women and Children's Health, Faculty of Life Sciences and Medicine, School of Life Course Sciences, King's College London, London, United Kingdom

7 - Department of Developmental Neuroscience, IRCCS, Fondazione Stella Maris, Pisa, Italy

8 - Department of Clinical and Experimental Medicine, University of Pisa, Pisa, Italy

9 - Department of Child and Adolescent Psychiatry and Psychology, Institute of Neurosciences, Hospital Clínic Universitari, Barcelona, Spain

10 - Department of Psychiatry, School of Medicine, Trinity College Dublin, Dublin, Ireland

11 - Center of Neurodevelopmental Disorders (KIND), Department of Women's and Children's Health, Centre for Psychiatry Research, Karolinska Institutet & Region Stockholm, Stockholm, Sweden

12 - Child and Adolescent Psychiatry, Stockholm Health Care Services, Region Stockholm, Stockholm, Sweden

13 - Curtin Autism Research Group, Curtin School of Allied Health, Curtin University, Perth, Australia

14 - Epilepsy Unit, Scientific Institute IRCCS E. Medea Conegliano, Treviso, Italy

15 - Centre for Psychiatry Research and Centre for Cognitive and Computational Neuropsychiatry (CCNP), Department of Clinical Neuroscience, Karolinska Institutet & Stockholm Health Care Services, Region Stockholm, Sweden

16 - Psykiatri Affektiva, Sahlgrenska University Hospital, Gothenburg, Sweden

17 - Institut d´Investigacions Biomèdiques August Pi i Sunyer (IDIBAPS), Barcelona, Spain

18 - Centro de Investigación Biomédica en Red de Salud Mental (CIBERSAM), Spain

For peer review only

19 - Department of Medicine, Institute of Neuroscience, University of Barcelona, Barcelona, Spain

20 - CIBIT, ICNAS, Faculty of Medicine, University of Coimbra, Coimbra, Portugal

21 - Institute of Human Genetics, University Hospital Heidelberg, Heidelberg, Germany

22 - Child and Adolescent Psychiatry Department, Robert Debre Hospital, APHP, Université Paris Cité, Paris, France

23 - Unit of Neurology and Clinical Neurophysiopathology, Oasi Research Institute-IRCCS, Troina, Italy

24 - Psychiatry Department, Centro Hospitalar e Universitário de Coimbra, Coimbra, Portugal

25 - Coimbra Institute for Biomedical Imaging and Translational Research, University of Coimbra, Coimbra, Portugal

26 - Institute of Psychological Medicine, Faculty of Medicine, University of Coimbra, Coimbra, Portugal

27 – Department of Forensic and Neurodevelopmental Sciences, Institute of Psychiatry, Psychology & Neuroscience, King's College London, London, United Kingdom

28 - Behavioural and Developmental Clinical Academic Group, South London and Maudsley NHS Foundation Trust, London, United Kingdom

29 - CIBIT - Coimbra Institute for Biomedical Imaging and Translational Research, University of Coimbra, Coimbra, Portugal

30 - ICNAS - Institute of Nuclear Sciences Applied to Health, University of Coimbra, Coimbra, Portugal

31 - Pediatric Neurology Department, Hospital Sant Joan de Déu, Institut de Recerca Sant Joan de Déu, Esplugues de Llobregat, Barcelona, Spain

For peer review only

For peer review only - http://bmjopen.bmj.com/site/about/guidelines.xhtml

32 - Astrid Lindgren Children's Hospital, Karolinska University Hospital, Region Stockholm, Stockholm, Sweden

33 - Neurological Sciences and Rehabilitation Medicine Scientific Area - Bambino Gesù Children's Hospital, Rome, Italy

34 – University Clinic of Pediatrics, Faculty of Medicine, University of Coimbra, 3000-602 Coimbra, Portugal

35 - Child Developmental Center and Research and Clinical Training Center, Pediatric Hospital, Centro Hospitalar e Universitário de Coimbra (CHUC), 3000-602 Coimbra, Portugal

36 - Cure Sanfilippo Foundation

37 - Dup15q Alliance

38 - Roche Pharma Research and Early Development, Roche Innovation Center Basel, Basel, Switzerland

39 - South London and Maudsley NHS Foundation Trust (SLaM), London SE5 8AZ, UK

40 - Institute for Translational Neurodevelopment, Institute of Psychiatry, Psychology & Neuroscience, King's College London, London WC2R 2LS, UK

41 - Department of Psychiatry at The Hospital for Sick Children

42 - Child and Youth Division Centre for Addiction and Mental Health, CAMH

43 - Department of Psychiatry, Temerty Faculty of Medicine, University of Toronto

44 - Génétique Humaine et Fonctions Cognitives, Institut Pasteur, UMR3571 CNRS, Université de Paris Cité, IUF, Paris, France

45 - F. Hoffman-La Roche Ltd, Basel, Switzerland

For peer review only

Corresponding author

Dr Alexandra Lautarescu, Department of Psychology, Institute of Psychiatry, Psychology & Neuroscience, King's College London, Alexandra.Lautarescu@kcl.ac.uk

For peer review only

For peer review only - http://bmjopen.bmj.com/site/about/guidelines.xhtml

ABSTRACT

Introduction: Autism is a common neurodevelopmental condition with a complex genetic aetiology that includes contributions from monogenic and polygenic factors. Many autistic people have unmet healthcare needs that could be served by genomics-informed research and clinical trials. The primary aim of the European Autism GEnomics Registry (EAGER) is to establish a registry of participants with a diagnosis of autism or an associated rare genetic condition who have undergone whole-genome sequencing. The registry can facilitate recruitment for future clinical trials and research studies, based on genetic, clinical, and phenotypic profiles, as well as participant preferences. The secondary aim of EAGER is to investigate the association between mental and physical health characteristics and participants' genetic profiles.

Methods and analysis*:* EAGER is a European multisite cohort study and registry and is part of the AIMS-2-TRIALS consortium. EAGER was developed with input from the AIMS-2-TRIALS Autism Representatives and representatives from the rare genetic conditions community. 1,500 participants with a diagnosis of autism or an associated rare genetic condition will be recruited at 13 sites across 8 countries. Participants will give a blood or saliva sample for whole-genome sequencing and answer a series of online questionnaires. Participants may also consent for the study to access pre-existing clinical data. Participants will be added to the EAGER registry. Data will be shared via the Autism Sharing Initiative, a new international collaboration aiming to create a federated system for autism data sharing.

Ethics and dissemination: EAGER has received full ethical approval from ethics committees in the UK (REC 23/SC/0022), Germany (S-375/2023), Portugal (CE-085/2023) and Spain (HCB/2023/0038, PIC-164-22). Approvals are in the process of being obtained from committees in Italy, Sweden, Ireland, and France. Findings will be disseminated via scientific publications and conferences, but also beyond to participants and the wider community (e.g., the AIMS-2-TRIALS website, stakeholder meetings, newsletters).

For peer review only - http://bmjopen.bmj.com/site/about/guidelines.xhtml

STRENGHTS AND LIMITATIONS OF THIS STUDY

- Data from full genotyping through whole-genome sequencing will be combined with mental and physical health data and participant research priorities
- The EAGER sample (n=1,500), although relatively small for genetic analyses, will include a substantial proportion (around one third) of participants with a rare genetic condition, ensuring that heterogeneous presentations across the autism spectrum are captured
- The EAGER registry will improve the speed, efficiency, and impact of research studies and clinical trials across Europe with a culturally diverse cohort of re-contactable participants, and shared data through the Autism Sharing Initiative
- EAGER was developed with input from the AIMS-2-TRIALS Autism Representatives and representatives from the rare genetic conditions community
- Phenotypic data are collected only via self/informant-report questionnaires and not direct clinical assessments

For peer review only - http://bmjopen.bmj.com/site/about/guidelines.xhtml

1. INTRODUCTION

Autism is a neurodevelopmental condition diagnosed on the basis of differences in social communication and interaction, sensory processing, restricted and repetitive behaviours, and intense interests (1,2), with a prevalence of around 1-2% of the general population (3). Autism is associated with a very large number of common genetic variants of small effect (4), as well as being associated with several rare genetic conditions such as Dup15q syndrome, Rett syndrome, tuberous sclerosis complex, and Angelman syndrome, where autism prevalence is as high as 30-93% (5,6).

Many autistic people have co-occurring mental and physical health conditions that impact quality of life, such as depression, anxiety, gastrointestinal symptoms, and epilepsy (7–12). For some autistic people and those with associated rare genetic conditions, many of these conditions can be severe and life-limiting, including respiratory and cardiac disorders in Rett syndrome (13,14), hyperphagia in Prader-Willi syndrome (15), and tumour growth in tuberous sclerosis complex (16). This makes the development of treatments and support for these populations a crucial area of research. Further, some autistic people may find aspects of being autistic distressing and disabling and may want help with these (17–20). All autistic people have the right to evidence-based care and support for aspects that are detrimental to their quality of life and wellbeing (21). Despite this, there is currently a paucity of personalised evidence-based care and support for autistic people across the spectrum (22,23), and polypharmacy (i.e., taking multiple medications) for various co-occurring symptoms is commonplace (24).

For peer review only - http://bmjopen.bmj.com/site/about/guidelines.xhtml

With a heritability of around 80%, autism has strong genetic influences (e.g., 25,26). However, to date, there is no known gene that, when mutated, increases the likelihood of an autism diagnosis without also increasing the likelihood of intellectual disability or other neurodevelopmental conditions (27). Consequently, genetic research is an important biomedical tool in the study of autism and associated rare genetic conditions, with benefits including personalised medicine, early identification of health risks, a better self-understanding, and a sense of community (28). To aid the development of evidence-based care for autistic people and those with associated rare genetic conditions, it is crucial to better understand genetic differences between syndromic forms of autism and autism without a known highly penetrant genetic influence. Further, by linking genetic mechanisms to treatment outcomes, autistic people and those with associated rare genetic conditions can be offered accurate information about what difficulties they may face both presently and in the future, and what interventions may be effective for aspects of their lived experience that they want or need support with.

EAGER (European Autism GEnomics Registry) is a European multicentre study that aims to create a registry of 1,500 participants with a diagnosis of autism or a rare genetic condition associated with autism. Participants will give a biosample and whole-genome sequencing will be performed on extracted DNA; participants will also answer a series of questionnaires related to autism, quality of life, mental and physical health, and research preferences. Using extensive genetic and phenotypic data, EAGER will enable a better understanding of the genetic architecture of autism and associated rare genetic conditions, as well as the relationship between genetics and outcomes such as health, quality of life, and wellbeing.

The EAGER registry will allow participants to be recontacted for future research studies or clinical trials that may be suitable for them. This will facilitate research by streamlining and

For peer review only

accelerating recruitment across Europe, as well as bring together key research and clinical institutions, to the benefit of participants and their families. In line with tailoring evidence-based care to specific populations, clinical trials that are genetically-informed allow researchers to invite participants who are the most suited to, and may benefit most from, a treatment (29). Selective recruitment at the early stages of treatment development encourages the downstream development of personalised medicine.

This project aims to be a catalyst for future research and clinical trials in autism and associated rare genetic conditions in Europe. This will be achieved by connecting key sites and allowing research groups and pharmaceutical companies planning research studies or clinical trials to have access to a European registry of people who have consented to being recontacted. EAGER data will also be shared via the Autism Sharing Initiative (ASI), a global collaboration connecting autism data through federated mechanisms (30). The genetic, phenotypic, and clinical data collected in EAGER can therefore be analysed with other international autism datasets such as MSSNG (31), POND (32), LEAP (33), SPARK (34), and Searchlight (35), providing the opportunity to investigate key research questions.

For peer review only

2. METHODS AND ANALYSIS

2.1. Study design and population

EAGER is an international, multisite registry of adults and children with a diagnosis of autism or associated rare genetic conditions. Recruitment for EAGER will take place at 13 sites across 8 European countries (see figure 1).

Figure 1. EAGER collaborating centres

To be eligible, participants need to be over 2 years of age and have a diagnosed rare genetic condition associated with autism and/or have a diagnosis of autism (see figure 2). Decisions regarding which conditions and genetic variants to include have been made on the basis of published literature (36) and discussions with experts in the field (TB, LG), with consideration given to the penetrance, prevalence, and association with autism. For participants with a diagnosis of autism only, and where existing genetic information is available, recruitment will prioritise individuals within multiplex families and individuals with genetic variants associated with autism (see figure 2). Participants will not be excluded from the study based on any other characteristics such as co-occurring conditions, sex, or intellectual disability.

Figure 2. EAGER protocol summary

For peer review only - http://bmjopen.bmj.com/site/about/guidelines.xhtml

For peer review only

2.2. Patient and public involvement and ethical consultations

Genetic research can involve complex ethical challenges, making it crucial to prioritise ethical considerations and participatory research practices (21,28). Participatory research practices can help ensure that the interests of our participants are appropriately represented and that the study is beneficial to its participants and the wider community. EAGER was developed with crucial input and feedback from community representatives via dedicated working groups. Our autism working group consists of AIMS-2-TRIALS Autism Representatives: autistic people and family members or carers of autistic people. Our rare genetic conditions working group consists of parents/carers of people with rare genetic conditions and/or representatives from European rare genetic conditions associations. As EAGER was conceptualised and funded prior to the establishment of our lived experience working groups, it is not a fully participatory research endeavour. This was discussed with and understood by all members of the working groups at the outset of our collaboration. Within these constraints, every effort was made to consult with our lived experience partners throughout the lifespan of this project, and feedback was integrated to the greatest degree possible.

The EAGER working groups have offered invaluable advice and feedback to guide the planning process of EAGER (see Box 1), either as part of working group meetings or through communication via email or contributions to collaborative documents. Open discussion within these groups was encouraged; as the groups included a variety of perspectives, there were naturally different solutions to questions. When no consensus was reached, the core research team made the final decision based on feasibility.

For peer review only - http://bmjopen.bmj.com/site/about/guidelines.xhtml

Box 1. Key examples of how patient and public involvement guided the EAGER study

- Refining the study rationale and clarifying perceived benefit of the study for autistic people
- Deciding on study name and logo
- Discussing ethical implications of the research, ensuring that appropriate safeguards are in place and that any limitations are transparently communicated to participants
- Ensuring that the information presented in study documents is clear, accessible, transparent, and sufficient to allow participants to make an informed decision about their participation
- Creating a “Data sharing Frequently Asked Questions” document to accompany the information sheet
- Developing different versions of information sheets tailored to the needs of our different participant groups (e.g., easy-read, adolescent information sheet)
- Ensuring that the consent forms are clear and transparent about what precisely a participant is consenting to
- Ensuring that the language used in study documents is appropriate and in line with community preferences
- Discussing the phenotypic measures used in the study and suggesting changes to the content/language of questionnaires
- Sharing all key study documents for feedback prior to submission for ethical approval
- Clearly stating that EAGER is against eugenics / prenatal testing for autism on participant-facing documents
- Detailing exactly what analyses will be performed on participant data and making it clear that no further analyses will occur without reconsenting
- Clarifying that analyses performed on EAGER data will need to be approved by a committee before they can begin
- Clarifying in the ethics documents that not all decisions were made based on consensus
- Adding a disclaimer in the protocol/ethics with regards to the fact that citing papers does not represent an endorsement of the language/aims of those papers or of the authors

For peer review only

For peer review only - http://bmjopen.bmj.com/site/about/guidelines.xhtml

Further, we conducted consultation with other stakeholders, ethical advisors, and additional working groups within the consortium, in order to evaluate risks and potentially sensitive implications of this research. We hope that the aforementioned groups will guide EAGER in the most beneficial direction for its participants and the wider community. These groups will continue to be consulted throughout the lifetime of the project.

2.3. Participant recruitment, information sheets, and informed consent

Potential participants will be approached by representatives at each local site. Each site will screen and recruit participants based upon recontact of participants from prior research studies or prior/current patients that fit the inclusion criteria.

Written informed consent will be required for participation in the study and participants will be provided with information sheets tailored to their age and levels of intellectual disability (see figure 2 for details on documents for specific participant groups). A personal consultee will be asked to consent on behalf of individuals who are deemed to not have capacity to consent following a capacity to consent assessment. Due to the high prevalence of co-occurring intellectual disability in people with rare genetic conditions, these may make up a significant proportion of the overall sample. Parental consent will be required for those under the legal age of consent (e.g., 16 years old in the UK).

To take part in EAGER, participants must consent to core aspects of the study: giving a blood or saliva sample (or permission to use a pre-existing sample), completing questionnaires, being added to the registry, and data sharing.

For peer review only - http://bmjopen.bmj.com/site/about/guidelines.xhtml

2.4. Biological sample collection

Participants will give a blood or saliva sample, depending on preference and appropriateness. The Oragene®•DNA self-collection kits, as well as assisted collection kits for those unable to collect their own sample, will be used for saliva collection. If existing blood or saliva samples are available and have been stored appropriately, they can be used in lieu of a new sample, with participant consent.

2.5. Measures

Following collection of the biological sample, participants will be given access to an online form with a series of questionnaires, focussing on autism, rare genetic conditions, co-occurring physical and mental health conditions, opinions on research priorities, and quality of life and wellbeing (see table 1). These will be a mixture of validated and non-validated questionnaires that have been adapted for EAGER, and have been chosen to overlap with other major initiatives or studies both within AIMS-2-TRIALS (37,38) and externally, such as MSSNG (31), POND (32), LEAP (33), SPARK (34), and Simon's Searchlight (35). All questionnaires have been translated into the local language of each site from English. The online questionnaires will be completed by participants answering on behalf of themselves, or by parents and consultees answering on behalf of the participant.

For peer review only

For peer review only - http://bmjopen.bmj.com/site/about/guidelines.xhtml

Table 1. EAGER questionnaires

Domain	Measure	Description	Respondents
**Autism traits**	Social Responsiveness Scale (SRS (39))	65 items: Autism traits (including social awareness, cognition, communication, motivation, and repetitive interests and behaviours)	Adult self-report
	Social Communication Questionnaire (SCQ, (40))	40 items: Autism traits (including communication skills and social functioning)	Parent or consultee report for child or adult
**Mental health**	Strengths and Difficulties Questionnaire (SDQ, (41))	25 items: General mental health across five scales (conduct problems, hyperactivity, emotional problems, peer problems, prosocial behaviour)	Parent or consultee report for adult or child
	Patient Health Questionnaire-9 (PHQ-9, (42))	9 items: Core features of major depressive disorder	Adult self-report
	Patient Health Questionnaire-A (PHQ-A, (43))	9 items: Core features of major depressive disorder*	Adolescent (16 and 17 years) self-report
	Generalised Anxiety Disorder-7 (GAD-7, (44))	7 items: Core features of generalised anxiety disorder	Adult self-report
**Quality of life**	World Health Organization Quality of Life-BREF (WHO-QOL-BREF, (45))	26 items: Quality of life across four domains (physical health, psychological health, social relationships, environment)	Adult self-report
	Cantrill Ladder (46)	1 item: Subjective quality of life (10 point scale from “worst possible life” to “best possible life”)	Adult self-report. Parent or consultee report for child or adult.

For peer review only

For peer review only - http://bmjopen.bmj.com/site/about/guidelines.xhtml

**Mental wellbeing**	Warwick-Edinburgh Mental Wellbeing Scales (WEMWBS, (47))	14 items: Mental wellbeing and health	Adult self-report. Parent or consultee self-report[+]
**Restrictive and repetitive behaviours**	Adult Routines inventory (ARI, (48))<br><br>Repetitive Behaviours Scale-Revised (RBS-R, (49))	55 items: Restrictive and repetitive behaviours<br><br>43: Assessing restrictive and repetitive behaviours	Adult self-report<br><br>Parent or consultee report for child or adult
**ADHD**	Conners' Adult ADHD Rating Scales (CAARS, (50))<br><br>ADHD Rating Scale (51))	26 items: Core features of ADHD<br><br>18 items: Core features of ADHD	Adult self-report<br><br>Parent report for child Consultee report for adult^
**Demographics**	Non-validated questionnaire	16-25 items: Demographic characteristics	Adult self-report. Parent or consultee report for child or adult.
**Co-occurring conditions**	Non-validated questionnaire (adapted from Simons Searchlight (35))	Items assessing 168 diagnosed and self-diagnosed psychiatric, neurodevelopmental, and physical health conditions.	Adult self-report. Parent or consultee report for child or adult.
**Medications**	Non-validated questionnaire	1 item: Number of medications taken by participant per day	Adult self-report. Parent or consultee report for child or adult.
**General functioning**	Non-validated questionnaire (adapted from (52))	7-11 items: Seizures, gross motor skills, language and communication, intellectual disability, support needs, sleep	Adult self-report. Parent or consultee report for child or adult.

For peer review only - http://bmjopen.bmj.com/site/about/guidelines.xhtml

**Voice of the community**	Non-validated questionnaire (adapted from Simon Searchlight (35))	7-13 items: Priorities for research	Adult self-report. Parent or consultee report for child or adult.

* Additional questions and assessment of suicidality in the original measure were removed for the purpose of this study and only the first core 9 items were included.

^ The ADHD rating scale was adapted for use in EAGER to make it appropriate for adults and remove child-specific language.

[+] Administered to parents and caregivers in relation to their own mental wellbeing.

2.6. *Data analysis*

EAGER will collect genetic and phenotypic data, which can be analysed both alone and in conjunction with other data collected within AIMS-2-TRIALS and/or shared via the ASI. Research questions will be decided in collaboration with our community working groups. Examples include identifying genetic variants that are associated with aspects of the core autism phenotype or conditions such as epilepsy or major depressive disorder, and identifying the association between genetic variants and/or polygenic scores and traits such as quality of life, levels of cognitive ability and executive function. The sample size of this study (n=1,500), which was driven by the available budget, is moderate-to-large in the field and will be significantly enhanced via the planned data sharing and integration.

For peer review only

3. DATA SHARING AND EAGER REGISTRY

3.1. Data sharing

Currently, funding bodies and participant groups have identified the need for research groups to share their data to maximise the value of each research contribution, pool data to address research questions that require a larger number of participants, or to carry out meta-analyses (53). For this reason, EAGER data will be shared via established AIMS-2-TRIALS data sharing mechanisms as well as being directly implemented into the ASI as detailed below. Rigorous safeguards will be implemented for access to data via established AIMS-2-TRIALS mechanisms and via the ASI platform (30).

Researchers will not be able to access EAGER data without approval from a data access committee, which will consist of autistic and non-autistic people, researchers, and members of the rare genetic community.

3.1.1. Autism Sharing Initiative (ASI)

The ASI is a new collaboration working to create the first federated, global network for sharing genomic and clinical data in autism research. The ASI is led by software company DNAStack and involves other collaborators such as Autism Speaks, Roche, and the Ontario Brain Institute (please see (54) for the full list). This is an ongoing project and new collaborators may be added.

Data sharing within the ASI will be built upon decentralised, federated data sharing principles. Data federation allows researchers to search, access, and analyse data that

For peer review only

386 reside in different locations and are connected via a network, without the need for data to be
387 moved between institutions/researchers.

388

389 Data federation allows researchers to interrogate and analyse data hosted in different
390 locations, in compliance with data regulations and improving privacy and security. A
391 federated data network involves connecting individual datasets, which remain in their
392 protected local environments (i.e., at the research institution where the data was collected),
393 across one cloud-based network. To our knowledge, the ASI carries no additional risk to
394 participant re-identification than traditional data sharing. The ASI software is built upon
395 security standards developed by the Global Alliance for Genomics and Health (55), and
396 regularly updated in order to ensure that data are safeguarded.

397

398 Within the ASI, data will include genomic, multi-omic, medical, and clinical data from
399 international autism research sources. With participant consent, EAGER data (genetic data,
400 data from online questionnaires, and existing data held at local sites) will be shared through
401 the ASI platform with any explicit identifiers removed.

402

403 ***3.2. EAGER registry***

404 The EAGER registry will allow participants to be contacted for future clinical trials and
405 research studies. Researchers viewing shared participant data will be able to contact the
406 coordinating site (King's College London) to request specific participant groups based upon
407 their desired criteria without access to participant personal information. King's College
408 London will pass these requests onto the relevant local sites, who can send information
409 about the study or clinical trial to participants.

410

For peer review only

4. ETHICS AND DISSEMINATION

4.1. Ethics

EAGER has received full ethical approval from ethics committees in the UK (REC 23/SC/0022), Germany (S-375/2023), Portugal (CE-085/2023) and Spain (HCB/2023/0038, PIC-164-22). Approvals are in the process of being obtained from ethics committees in Italy, Sweden, Ireland, and France**.** Please refer to the above section "Patient and Public Involvement and Ethics Consultation" for detail on ethics and discussions within our community working groups.

4.2. Dissemination

We plan to disseminate our findings widely via academic avenues, such as publications in peer-reviewed major international scientific journals and conferences. In addition, we will disseminate beyond academic avenues to ensure that our findings reach participants and the wider communities, including the AIMS-2-TRIALS website (www.aims2trials.eu), stakeholder meetings, and newsletters. As part of the overall AIMS-2-TRIALS project, we have access to a European network including researchers, clinicians, and families within the autism community. This network will also provide a forum for dissemination of research findings and training opportunities.

Disclaimer: Citing papers does not represent an endorsement of the language used in these papers, nor of the aims of the research or researchers.

For peer review only - http://bmjopen.bmj.com/site/about/guidelines.xhtml

REFERENCES

1. Lord C, Brugha TS, Charman T, Cusack J, Dumas G, Frazier T, et al. Autism spectrum disorder. Nat Rev Dis Primers. 2020 Jan 16;6(1):1–23.

2. Kodak T, Bergmann S. Autism Spectrum Disorder: Characteristics, Associated Behaviors, and Early Intervention. Pediatr Clin North Am. 2020 Jun;67(3):525–35.

3. Zeidan J, Fombonne E, Scorah J, Ibrahim A, Durkin MS, Saxena S, et al. Global prevalence of autism: A systematic review update. Autism Res. 2022 May;15(5):778–90.

4. Willsey HR, Willsey AJ, Wang B, State MW. Genomics, convergent neuroscience and progress in understanding autism spectrum disorder. Nat Rev Neurosci. 2022 Jun;23(6):323–41.

5. Richards C, Jones C, Groves L, Moss J, Oliver C. Prevalence of autism spectrum disorder phenomenology in genetic disorders: a systematic review and meta-analysis. The Lancet Psychiatry. 2015 Oct 1;2(10):909–16.

6. Lusk L, Vogel-Farley V, DiStefano C, Jeste S. Maternal 15q Duplication Syndrome. In: Adam MP, Ardinger HH, Pagon RA, Wallace SE, Bean LJ, Gripp KW, et al., editors. GeneReviews® [Internet]. Seattle (WA): University of Washington, Seattle; 1993 [cited 2022 May 9]. Available from: http://www.ncbi.nlm.nih.gov/books/NBK367946/

7. Lai MC, Kassee C, Besney R, Bonato S, Hull L, Mandy W, et al. Prevalence of co-occurring mental health diagnoses in the autism population: a systematic review and meta-analysis. Lancet Psychiatry. 2019 Oct;6(10):819–29.

8. Tye C, Runicles AK, Whitehouse AJO, Alvares GA. Characterizing the Interplay Between Autism Spectrum Disorder and Comorbid Medical Conditions: An Integrative Review. Front Psychiatry. 2018;9:751.

For peer review only - http://bmjopen.bmj.com/site/about/guidelines.xhtml

9. van Heijst BF, Deserno MK, Rhebergen D, Geurts HM. Autism and depression are connected: A report of two complimentary network studies. Autism. 2020 Apr;24(3):680–92.

10. Hollocks MJ, Lerh JW, Magiati I, Meiser-Stedman R, Brugha TS. Anxiety and depression in adults with autism spectrum disorder: a systematic review and meta-analysis. Psychological Medicine. 2019 Mar;49(4):559–72.

11. Wang J, Ma B, Wang J, Zhang Z, Chen O. Global prevalence of autism spectrum disorder and its gastrointestinal symptoms: A systematic review and meta-analysis. Front Psychiatry. 2022 Aug 23;13:963102.

12. Besag FM. Epilepsy in patients with autism: links, risks and treatment challenges. Neuropsychiatr Dis Treat. 2017 Dec 18;14:1–10.

13. Acampa M, Guideri F. Cardiac disease and Rett syndrome. Arch Dis Child. 2006 May;91(5):440–3.

14. Katz DM, Dutschmann M, Ramirez JM, Hilaire G. Breathing Disorders in Rett Syndrome: Progressive Neurochemical Dysfunction in the Respiratory Network after Birth. Respir Physiol Neurobiol. 2009 Aug 31;168(1–2):101–8.

15. Driscoll DJ, Miller JL, Cassidy SB. Prader-Willi Syndrome. In: Adam MP, Mirzaa GM, Pagon RA, Wallace SE, Bean LJ, Gripp KW, et al., editors. GeneReviews® [Internet]. Seattle (WA): University of Washington, Seattle; 1993 [cited 2023 Sep 18]. Available from: http://www.ncbi.nlm.nih.gov/books/NBK1330/

16. Islam MP. Tuberous Sclerosis Complex. Semin Pediatr Neurol. 2021 Apr;37:100875.

17. Schaaf RC, Toth-Cohen S, Johnson SL, Outten G, Benevides TW. The everyday routines of families of children with autism: examining the impact of sensory processing difficulties on the family. Autism. 2011 May;15(3):373–89.

For peer review only

For peer review only - http://bmjopen.bmj.com/site/about/guidelines.xhtml

18. Green D, Chandler S, Charman T, Simonoff E, Baird G. Brief Report: DSM-5 Sensory Behaviours in Children With and Without an Autism Spectrum Disorder. J Autism Dev Disord. 2016 Nov 1;46(11):3597–606.

19. Lai MC, Anagnostou E, Wiznitzer M, Allison C, Baron-Cohen S. Evidence-based support for autistic people across the lifespan: maximising potential, minimising barriers, and optimising the person-environment fit. Lancet Neurol. 2020 May;19(5):434–51.

20. Lord C, Charman T, Havdahl A, Carbone P, Anagnostou E, Boyd B, et al. The Lancet Commission on the future of care and clinical research in autism. The Lancet. 2022 Jan;399(10321):271–334.

21. Heraty S, Lautarescu A, Belton D, Boyle A, Cirrincione P, Doherty M, et al. Bridge-building between communities: Imagining the future of biomedical autism research. Cell. 2023 Aug 31;186(18):3747–52.

22. Sandbank M, Bottema-Beutel K, Crowley S, Cassidy M, Dunham K, Feldman JI, et al. Project AIM: Autism intervention meta-analysis for studies of young children. Psychological Bulletin. 2020;146(1):1–29.

23. Braga LAM, Conte Filho CG, Mota FB. Future of genetic therapies for rare genetic diseases: what to expect for the next 15 years? Ther Adv Rare Dis. 2022 Jun 10;3:26330040221100840.

24. Feroe AG, Uppal N, Gutiérrez-Sacristán A, Mousavi S, Greenspun P, Surati R, et al. Medication Use in the Management of Comorbidities Among Individuals With Autism Spectrum Disorder From a Large Nationwide Insurance Database. JAMA Pediatrics. 2021 Sep 1;175(9):957–65.

For peer review only

25. Bai D, Yip BHK, Windham GC, Sourander A, Francis R, Yoffe R, et al. Association of Genetic and Environmental Factors With Autism in a 5-Country Cohort. JAMA Psychiatry. 2019 Oct 1;76(10):1035–43.

26. Tick B, Bolton P, Happé F, Rutter M, Rijsdijk F. Heritability of autism spectrum disorders: a meta-analysis of twin studies. Journal of Child Psychology and Psychiatry. 2016;57(5):585–95.

27. Myers SM, Challman TD, Bernier R, Bourgeron T, Chung WK, Constantino JN, et al. Insufficient Evidence for "Autism-Specific" Genes. Am J Hum Genet. 2020 May 7;106(5):587–95.

28. Natri HM, Chapman CR, Heraty S, Dwyer P, Walker N, Kapp SK, et al. Ethical challenges in autism genomics: Recommendations for researchers. European Journal of Medical Genetics. 2023 Sep 1;66(9):104810.

29. Díaz-Caneja CM, State MW, Hagerman RJ, Jacquemont S, Marín O, Bagni C, et al. A white paper on a neurodevelopmental framework for drug discovery in autism and other neurodevelopmental disorders. Eur Neuropsychopharmacol. 2021 Jul;48:49–88.

30. DNAstack [Internet]. [cited 2023 Oct 8]. DNAstack launches Neuroscience AI to accelerate collaborative research and discovery, DNAstack. Available from: https://dnastack.com/news-neuroscience-ai-launch-announcement/

31. MSSNG [Internet]. [cited 2023 Sep 18]. Available from: https://research.mss.ng/

32. POND. Your Research Priorities [Internet]. Province of Ontario Neurodevelopmental Network. [cited 2023 Apr 11]. Available from: https://pond-network.ca/your-research-priorities/

For peer review only - http://bmjopen.bmj.com/site/about/guidelines.xhtml

33. Charman T, Loth E, Tillmann J, Crawley D, Wooldridge C, Goyard D, et al. The EU-AIMS Longitudinal European Autism Project (LEAP): clinical characterisation. Molecular Autism. 2017 Jun 23;8(1):27.

34. SPARK [Internet]. [cited 2023 Sep 18]. Available from: https://sparkforautism.org/

35. SFARI [Internet]. 2019 [cited 2023 Sep 18]. SFARI | Simons Searchlight. Available from: https://www.sfari.org/resource/simons-searchlight/

36. Rolland T, Cliquet F, Anney RJL, Moreau C, Traut N, Mathieu A, et al. Phenotypic effects of genetic variants associated with autism. Nat Med. 2023 Jul;29(7):1671–80.

37. AIMS-2-TRIALS - Autism Research For Europe [Internet]. 2018 [cited 2023 Oct 8]. Available from: https://www.aims-2-trials.eu/

38. Oakley BFM, Loth E, Jones EJH, Chatham CH, Murphy DG. Advances in the identification and validation of autism biomarkers. Nat Rev Drug Discov. 2022 Oct;21(10):697–8.

39. Constantino JN, Davis SA, Todd RD, Schindler MK, Gross MM, Brophy SL, et al. Validation of a Brief Quantitative Measure of Autistic Traits: Comparison of the Social Responsiveness Scale with the Autism Diagnostic Interview-Revised. J Autism Dev Disord. 2003 Aug 1;33(4):427–33.

40. (SCQ) Social Communication Questionnaire [Internet]. [cited 2023 Sep 18]. Available from: https://www.wpspublish.com/scq-social-communication-questionnaire.html

41. Goodman R. The Strengths and Difficulties Questionnaire: a research note. J Child Psychol Psychiatry. 1997 Jul;38(5):581–6.

42. Kroenke K, Spitzer RL, Williams JB. The PHQ-9: validity of a brief depression severity measure. J Gen Intern Med. 2001 Sep;16(9):606–13.

For peer review only - http://bmjopen.bmj.com/site/about/guidelines.xhtml

43. Johnson JG, Harris ES, Spitzer RL, Williams JBW. The patient health questionnaire for adolescents: validation of an instrument for the assessment of mental disorders among adolescent primary care patients. J Adolesc Health. 2002 Mar;30(3):196–204.

44. Spitzer RL, Kroenke K, Williams JBW, Löwe B. A Brief Measure for Assessing Generalized Anxiety Disorder: The GAD-7. Archives of Internal Medicine. 2006 May 22;166(10):1092–7.

45. WHOQOL - Measuring Quality of Life| The World Health Organization [Internet]. [cited 2023 Sep 18]. Available from: https://www.who.int/tools/whoqol

46. Cantril H. The pattern of human concerns. New Brunswick, N.J.: Rutgers University Press; 1966. 427 p.

47. Tennant R, Hiller L, Fishwick R, Platt S, Joseph S, Weich S, et al. The Warwick-Edinburgh Mental Well-being Scale (WEMWBS): development and UK validation. Health and Quality of Life Outcomes. 2007 Nov 27;5(1):63.

48. Evans DW, Uljarević M, Lusk LG, Loth E, Frazier T. Development of Two Dimensional Measures of Restricted and Repetitive Behavior in Parents and Children. Journal of the American Academy of Child & Adolescent Psychiatry. 2017 Jan 1;56(1):51–8.

49. Bodfish JW, Symons FJ, Parker DE, Lewis MH. Repetitive Behavior Scale–Revised [Internet]. 2014 [cited 2023 Sep 18]. Available from: http://doi.apa.org/getdoi.cfm?doi=10.1037/t17338-000

50. Macey KD. Conners' Adult ADHD Rating Scales (CAARS). Archives of Clinical Neuropsychology. 2003 May 1;18(4):431–7.

51. DuPaul GJ, Power TJ, Anastopoulos AD, Reid R. ADHD Rating Scale—IV: Checklists, norms, and clinical interpretation. New York, NY, US: The Guilford Press; 1998. viii, 79 p. (ADHD Rating Scale—IV: Checklists, norms, and clinical interpretation).

For peer review only - http://bmjopen.bmj.com/site/about/guidelines.xhtml

573 52. Keute M, Miller MT, Krishnan ML, Sadhwani A, Chamberlain S, Thibert RL, et al. Angelman syndrome genotypes manifest varying degrees of clinical severity and developmental impairment. Mol Psychiatry. 2021 Jul;26(7):3625–33.

576 53. Lombardo MV, Lai MC, Baron-Cohen S. Big data approaches to decomposing heterogeneity across the autism spectrum. Mol Psychiatry. 2019 Oct;24(10):1435–50.

578 54. Digital Supercluster [Internet]. [cited 2023 Oct 8]. Autism Sharing Initiative. Available from: https://www.digitalsupercluster.ca/projects/autism-sharing-initiative/

580 55. Global Alliance for Genomics and Health. Enabling responsible genomic data sharing for the benefit of human health [Internet]. 2023 [cited 2023 Apr 11]. Available from: https://www.ga4gh.org/

583

584

585 **AUTHOR CONTRIBUTIONS:**

586 **Conceptualisation:** MB, AL, SH, SD, PV, RP, AG, KVdB, EE, CO, VR, JT, BO, DM, LG, TB, CC, TC. **Funding acquisition:** DM, CC, TC**. Project administration:** MB, AL, DM, LG, TB, CC. TC. **Supervision:** AL, TC. **Visualisation:** MB, AL. **Data collection**: MB, AL, MA, RB, ABH, NB, SB, PB, JB, SC, RCE, MCB, JCF, PC, AD, RD, ME, MH, NM, GM, RM, CJM, SM, VM, APR, CPS, MS, KT, CT, FV, GO, BM, LG, TC. **Writing - original draft:** MB, AL, TC. **Writing - review and editing:** All authors

592

593 **FUNDING STATEMENT:**

594 This work has received funding from the Innovative Medicines Initiative 2 Joint Undertaking under grant agreement No 777394 for the project AIMS-2-TRIALS. This Joint Undertaking receives support from the European Union's Horizon 2020 research and innovation

For peer review only - http://bmjopen.bmj.com/site/about/guidelines.xhtml

programme and EFPIA and AUTISM SPEAKS, Autistica, SFARI and from Horizon Europe [grant agreement no. 101057385] and from Horizon Europe [grant agreement no. 101057385] and from UK Research and Innovation (UKRI) under the UK government's Horizon Europe funding guarantee [grant no.10039383] (R2D2-MH). The funders had no role in the design of the study; in the collection, analyses, or interpretation of data; in the writing of the manuscript, or in the decision to publish the results. Any views expressed are those of the author(s) and not necessarily those of the funders. In addition, SB receives funding from Swedish Research Council and Region Stockholm. CT receives funding from Epilepsy Research UK, Autistica, the Baily Thomas Charitable Fund and the Tuberous Sclerosis Association. LG receives funding from Horizon Europe: Risk and Resilience in Developmental Diversity and Mental Health (R2D2-MH). CPS receives funding from Foundation of Prader-Willi Research, European Joint Program for Rare Diseases, Illumina, German Children's Cancer Foundation, and the German Ministry of Education and Research. RB, RM, SC, BM receive funding from the Italian Ministry of Health (Ricerca Corrente and 5x1000 to IRCCS Fondazione Stella Maris). Genomic data utilised here will be sequenced in part/whole through the financial contribution of F Hoffmann La-Roche. This paper represents independent research part-funded by the NIHR Maudsley Biomedical Research Centre at South London and Maudsley NHS Foundation Trust and King's College London. The views expressed are those of the author(s) and not necessarily those of the NIHR or the Department of Health and Social Care.

COMPETING INTEREST STATEMENT:

In the past 3 years TC has served as a paid consultant to F. Hoffmann-La Roche Ltd. and Servier and has received royalties from Sage Publications and Guilford Publications. DM has received funding for a PhD studentship from Compass, and for consulting from Jaguar Therapeutics and Hoffman Le Roche. GM receives funding for an investigator-initiated study from Compass Pathways; no financial or other conflict of interest with the present study. SB

For peer review only - http://bmjopen.bmj.com/site/about/guidelines.xhtml

discloses that he has in the last 3 years acted as an author, consultant, or lecturer for Medice, Roche, and Linus Biotechnology. SB receives royalties for textbooks and diagnostic tools from Hogrefe, UTB, Ernst Reinhardt, Kohlhammer, and Liber, and is a partner at NeuroSupportSolutions International AB. CC is a full-time employee of Genentech and owns stocks or RSUs in Roche Holdings, Ltd. MA is the UK chief investigator for a trial sponsored by Roche (a phase II, randomised, double-blind, placebo-controlled, parallel group study to evaluate the safety, efficacy, and pharmacodynamics of 52 weeks of treatment with basmasanil in participants aged 2 to 14 years old with dup15q syndrome followed by a 2-year optional open-label extension). LB served on an advisory board to Kingdom therapeutics in 2022.

WORD COUNT: 2418

For peer review only

Location	Site	Role
Stockholm, Sweden	Karolinska Institutet	R
London, UK	King's College London	R, L
	Evelina London, Guy's & St Thomas NHS Foundation Trust	R
Dublin, Ireland	Trinity College Dublin	R
Paris, France	Assistance Publique-Hopitaux de Paris	R
	Commissariat a l'Energie Atomique et aux Energies Alternatives	S
	Institut Pasteur	S
Heidelberg, Germany	Heidelberg University Hospital	R
Treviso, Italy	Associazione La Nostra Famiglia Istituto Scientifico Eugenio Medea	R
Pisa, Italy	Fondazione Stella Maris	R
Rome, Italy	Ospedale Pediatrico Bambino Gesù	R
Troina, Italy	Associazione Oasi Maria SS. Onlus	R
Barcelona, Spain	Hospital Clinic de Barcelona & Fundacio Clinic per a la Recerca Biomedica	R
	Fundació Sant Joan de Déu	R
Coimbra, Portugal	University of Coimbra	R

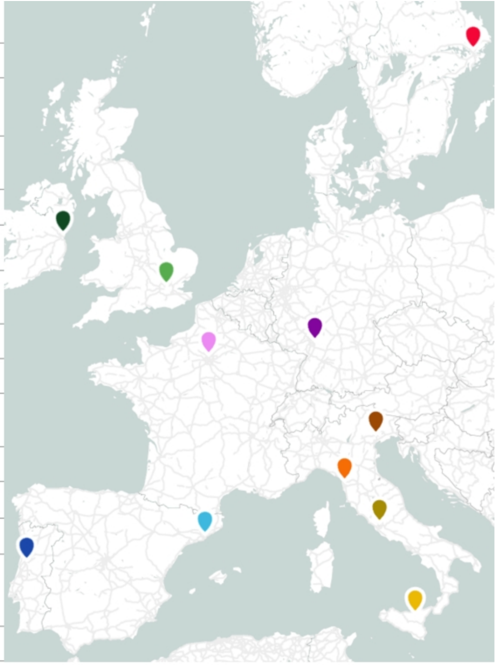

R=Recruitment, L=Lead centre, S=Sample storage, sequencing, analysis

Figure 1. EAGER collaborating centres

1500x1060mm (38 x 38 DPI)

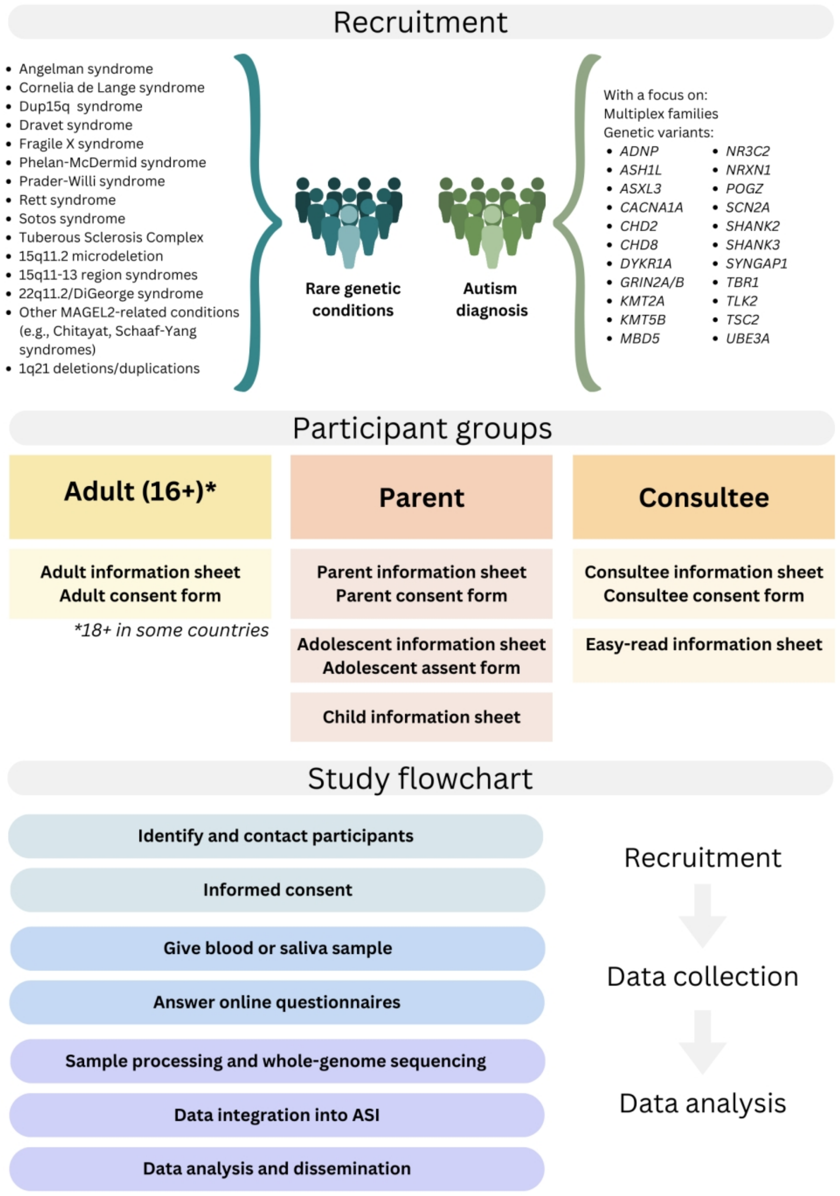

Figure 2. EAGER protocol summary

1060x1500mm (38 x 38 DPI)

BMJ Open

European Autism GEnomics Registry (EAGER): Protocol for a multicentre cohort study and registry

Journal:	*BMJ Open*
Manuscript ID	bmjopen-2023-080746.R1
Article Type:	Protocol
Date Submitted by the Author:	17-Apr-2024
Complete List of Authors:	Bloomfield, Madeleine; King's College London, Psychology, Institute of Psychiatry, Psychology & Neuroscience<br>Lautarescu, Alexandra; King's College London, Psychology, Institute of Psychiatry, Psychology & Neuroscience<br>Heraty, Síofra; Birkbeck University of London, Department of Psychological Sciences<br>Douglas, Sarah; Cambridge University, AIMS-2-TRIALS A-Reps<br>Violland, Pierre; Cambridge University, AIMS-2-TRIALS A-Reps<br>Plas, Roderik; Cambridge University, AIMS-2-TRIALS A-Reps<br>Ghosh, Anjuli; Cambridge University, AIMS-2-TRIALS A-Reps<br>Van den Bosch, Katrien; Cambridge University, AIMS-2-TRIALS A-Reps<br>Eaton, Eliza; Cambridge University, Autism Research Centre<br>Absound, Michael; Guy's and St Thomas' Hospitals NHS Trust, Department of Children's Neurosciences, Evelina London Children's Hospital; King's College London, Department of Women and Children's Health, Faculty of Life Sciences and Medicine, School of Life Course Sciences<br>Battini, Roberta ; IRCCS Fondazione Stella Maris, Department of Developmental Neuroscience; University of Pisa, Department of Clinical and Experimental Medicine<br>Blázquez Hinojosa, Ana ; Hospital Clinic Universitari Barcelona, Department of Child and Adolescent Psychiatry and Psychology, Institute of Neurosciences<br>Bolshakova, Nadia; Trinity College Dublin, Department of Psychiatry, School of Medicine<br>Bölte, Sven; Karolinska Institutet, Center of Neurodevelopmental Disorders (KIND), Department of Women's and Children's Health, Centre for Psychiatry Research; Stockholm Health Care Services, Child and Adolescent Psychiatry<br>Bonanni, Paolo; Scientific Institute IRCCS E. Medea Conegliano, Epilepsy Unit<br>Borg, Jacqueline ; Karolinska Institutet & Stockholm Health Care Services, Centre for Psychiatry Research and Centre for Cognitive and Computational Neuropsychiatry (CCNP), Department of Clinical Neuroscience; Sahlgrenska University Hospital, Department of Neuropsychiatry, Region Västra Götaland<br>Calderoni, Sara; IRCCS Fondazione Stella Maris, Department of Developmental Neuroscience; University of Pisa, Department of Clinical and Experimental Medicine<br>Calvo Escalona, Rosa; Hospital Clinic Universitari Barcelona, Department

of Child and Adolescent Psychiatry and Psychology, Institute of Neurosciences; Institut d'Investigacions Biomèdiques August Pi i Sunyer
Castelo-Branco, Miguel; University of Coimbra, Institute of Physiology, Faculty of Medicine; University of Coimbra, Coimbra Institute for Biomedical Imaging and Translational Research (CIBIT), Institute of Nuclear Sciences Applied to Health (ICNAS)
Castro-Fornieles, Josefina; Hospital Clinic Universitari Barcelona, Department of Child and Adolescent Psychiatry and Psychology, Institute of Neurosciences; Institut d'Investigacions Biomèdiques August Pi i Sunyer
Caro, Pilar; University Hospital Heidelberg, Institute of Human Genetics
Cliquet, Freddy; Institut Pasteur, Génétique Humaine et Fonctions Cognitives, UMR3571 CNRS
Danieli, Alberto; Scientific Institute IRCCS E. Medea Conegliano, Epilepsy Unit
Delorme, Richard; APHP, Child and Adolescent Psychiatry Department, Robert Debre Hospital
Elia, Maurizio; Oasi Research Institute-IRCCS, Unit of Neurology and Clinical Neurophysiopathology
Hempel, Maja; University Hospital Heidelberg, Institute of Human Genetics
Leblond, Claire; Institut Pasteur, Génétique Humaine et Fonctions Cognitives, UMR3571 CNRS
Madeira, Nuno; University of Coimbra, Institute of Physiology, Faculty of Medicine; Centro Hospitalar e Universitário de Coimbra EPE, Psychiatry Department
McAlonan, Grainne; King's College London, Department of Forensic and Neurodevelopmental Sciences, Institute of Psychiatry, Psychology, & Neuroscience; South London and Maudsley NHS Foundation Trust, Behavioural and Developmental Clinical Academic Group
Milone, Roberta; IRCCS Fondazione Stella Maris, Department of Developmental Neuroscience
Molloy, Ciara J.; Trinity College Dublin, Department of Psychiatry, School of Medicine
Mouga, Susana; University of Coimbra, Institute of Physiology, Faculty of Medicine
Montiel, Virginia; Institut de Recerca Sant Joan de Deu, Pediatric Neurology Department, Hospital Sant Joan de Déu
Pina Rodrigues, Ana; University of Coimbra, Institute of Physiology, Faculty of Medicine
Schaaf, Christian P.; University Hospital Heidelberg, Institute of Human Genetics
Serrano, Mercedes; Institut de Recerca Sant Joan de Deu, Pediatric Neurology Department, Hospital Sant Joan de Déu
Tammimies, Kristiina; Karolinska Institutet, Center of Neurodevelopmental Disorders (KIND), Department of Women’s and Children’s Health, Centre for Psychiatry Research; Karolinska University Hospital, Astrid Lindgren Children's Hospital
Tye, Charlotte; King's College London, Psychology, Institute of Psychiatry, Psychology & Neuroscience
Vigevano, Federico; Bambino Gesù Children's Hospital, Neurological Sciences and Rehabilitation Medicine Scientific Area; IRCCS San Raffaele, Paediatric Neurorehabilitation Department
Oliveira, Guiomar; University of Coimbra, Coimbra Institute for Biomedical Imaging and Translational Research (CIBIT), Institute of Nuclear Sciences Applied to Health (ICNAS); University of Coimbra, University Clinic of Pediatrics, Faculty of Medicine
Mazzone, Beatrice; IRCCS Fondazione Stella Maris, Department of Developmental Neuroscience
O’Neill, Cara ; Cure Sanfilippo Foundation
Pender, Julie; SYNGAP Research Fund

	Romero, Verena; Dup15q e.V.<br>Tillmann, Julian; Roche Innovation Center Basel, Roche Pharma Research and Early Development<br>Oakley, Bethany; King's College London, Department of Forensic and Neurodevelopmental Sciences, Psychology, Institute of Psychiatry, Psychology & Neuroscience<br>Murphy, Declan G. M.; King's College London, Department of Forensic and Neurodevelopmental Sciences, Psychology, Institute of Psychiatry, Psychology & Neuroscience; South London and Maudsley NHS Foundation Trust<br>Gallagher , Louise ; Trinity College Dublin, Department of Psychiatry, School of Medicine; The Hospital for Sick Children, SickKids Research Institute, Peter Gilgan Centre for Research and Learning<br>Bourgeron, Thomas; Institut Pasteur, Génétique Humaine et Fonctions Cognitives, UMR3571 CNRS<br>Chatham, Christopher; F Hoffmann-La Roche Ltd<br>Charman, Tony; King's College London, Department of Psychology, Institute of Psychiatry, Psychology & Neuroscience
**Primary Subject Heading**:	Genetics and genomics
Secondary Subject Heading:	Mental health
Keywords:	GENETICS, REGISTRIES, MENTAL HEALTH

SCHOLARONE™ Manuscripts

European Autism GEnomics Registry (EAGER): Protocol for a multicentre cohort study and registry

Authors:

Bloomfield, M.[1]*, Lautarescu, A.[1]*, Heraty, S.[2], Douglas, S.[3], Violland, P.[3], Plas, R.[3], Ghosh, A.[3], Van den Bosch, K.[3], Eaton, E.[4], Absoud, M.[5,6], Battini, R.[7,8], Blázquez Hinojosa, A.[9], Bolshakova, N.[10], Bolte, S.[11,12,13], Bonanni, P.[14], Borg, J.[15,16,17], Calderoni, S.[7,8], Calvo Escalona, R.[9,18,19,20], Castelo-Branco, M.[21,22], Castro-Fornieles, J.[9,18,19,20], Caro P.[23], Cliquet, F.[24], Danieli, A.[14], Delorme, R.[25], Elia, M.[26], Hempel, M.[23], Leblond, C. S.[24], Madeira, N.[21,27,28], McAlonan, G.[29,30], Milone, R.[7], Molloy, C. J.[10], Mouga, S.[21], Montiel, V.[31], Pina Rodrigues, A.[21], Schaaf, C. P.[23], Serrano, M.[31], Tammimies, K.[11,32], Tye, C.[1], Vigevano, F.[33,34], Oliveira, G.[22,35,36], Mazzone, B.[7], O'Neill, C.[37], Pender, J.[38], Romero, V.[39], Tillmann, J.[40], Oakley, B.[29], Murphy, D.[29,41,42], Gallagher, L.[10,43,44,45], Bourgeron, T.[24], Chatham, C.[46], Charman, T.[1]

* Indicates equal contribution

Affiliations:

1 - Department of Psychology, Institute of Psychiatry, Psychology & Neuroscience, King's College London, London, United Kingdom

2 - Department of Psychological Sciences, University of London, Birkbeck, London, United Kingdom

3 - AIMS-2-Trials A-Reps, University of Cambridge, Cambridge, United Kingdom

4 - Autism Research Centre, University of Cambridge, Cambridge, United Kingdom

5 - Department of Children's Neurosciences, Evelina London Children's Hospital, Guy's and St Thomas' NHS Foundation Trust, London, United Kingdom

For peer review only - http://bmjopen.bmj.com/site/about/guidelines.xhtml

6 - Department of Women and Children's Health, Faculty of Life Sciences and Medicine, School of Life Course Sciences, King's College London, London, United Kingdom

7 - Department of Developmental Neuroscience, IRCCS, Fondazione Stella Maris, Pisa, Italy

8 - Department of Clinical and Experimental Medicine, University of Pisa, Pisa, Italy

9 - Department of Child and Adolescent Psychiatry and Psychology, Institute of Neurosciences, Hospital Clínic Universitari, Barcelona, Spain

10 - Department of Psychiatry, School of Medicine, Trinity College Dublin, Dublin, Ireland

11 - Center of Neurodevelopmental Disorders (KIND), Department of Women's and Children's Health, Centre for Psychiatry Research, Karolinska Institutet & Region Stockholm, Stockholm, Sweden

12 - Child and Adolescent Psychiatry, Stockholm Health Care Services, Region Stockholm, Stockholm, Sweden

13 - Curtin Autism Research Group, Curtin School of Allied Health, Curtin University, Perth, Australia

14 - Epilepsy Unit, Scientific Institute IRCCS E. Medea Conegliano, Treviso, Italy

15 - Centre for Psychiatry Research and Centre for Cognitive and Computational Neuropsychiatry (CCNP), Department of Clinical Neuroscience, Karolinska Institutet & Stockholm Health Care Services, Region Stockholm, Sweden

16 - Region Västra Götaland, Sahlgrenska University Hospital, Dept of Neuropsychiatry, Gothenburg, Sweden

For peer review only - http://bmjopen.bmj.com/site/about/guidelines.xhtml

17 - Department of Psychiatry and Neurochemistry, Institute of Neuroscience and Physiology, Sahlgrenska Academy at The University of Gothenburg, Gothenburg, Sweden.

18 - Institut d´Investigacions Biomèdiques August Pi i Sunyer (IDIBAPS), Barcelona, Spain

19 - Centro de Investigación Biomédica en Red de Salud Mental (CIBERSAM), Spain

20 - Department of Medicine, Institute of Neuroscience, University of Barcelona, Barcelona, Spain

21 – Institute of Physiology, Faculty of Medicine, University of Coimbra, Coimbra, Portugal

22 - Coimbra Institute for Biomedical Imaging and Translational Research (CIBIT), Institute of Nuclear Sciences Applied to Health (ICNAS), University of Coimbra, Coimbra, Portugal

23 - Institute of Human Genetics, University Hospital Heidelberg, Heidelberg, Germany

24 - Génétique Humaine et Fonctions Cognitives, Institut Pasteur, UMR3571 CNRS, Université de Paris Cité, IUF, Paris, France

25 - Child and Adolescent Psychiatry Department, Robert Debre Hospital, APHP, Université Paris Cité, Paris, France

26 - Unit of Neurology and Clinical Neurophysiopathology, Oasi Research Institute-IRCCS, Troina, Italy

27 - Psychiatry Department, Centro Hospitalar e Universitário de Coimbra, Coimbra, Portugal

For peer review only

For peer review only - http://bmjopen.bmj.com/site/about/guidelines.xhtml

28 - Institute of Psychological Medicine, Faculty of Medicine, University of Coimbra, Coimbra, Portugal

29 – Department of Forensic and Neurodevelopmental Sciences, Institute of Psychiatry, Psychology & Neuroscience, King's College London, London, United Kingdom

30 - Behavioural and Developmental Clinical Academic Group, South London and Maudsley NHS Foundation Trust, London, United Kingdom

31 - Pediatric Neurology Department, Hospital Sant Joan de Déu, Institut de Recerca Sant Joan de Déu, Esplugues de Llobregat, Barcelona, Spain

32 - Astrid Lindgren Children's Hospital, Karolinska University Hospital, Region Stockholm, Stockholm, Sweden

33 - Neurological Sciences and Rehabilitation Medicine Scientific Area - Bambino Gesù Children's Hospital, Rome, Italy

34 – Paediatric Neurorehabilitation Department, IRCCS San Raffaele, Rome, Italy

35 – University Clinic of Pediatrics, Faculty of Medicine, University of Coimbra, 3000-602 Coimbra, Portugal

36 - Child Developmental Center and Research and Clinical Training Center, Pediatric Hospital, Centro Hospitalar e Universitário de Coimbra (CHUC), 3000-602 Coimbra, Portugal

37 - Cure Sanfilippo Foundation

38 – SYNGAP Research Fund

39 - Dup15q e. V.

40 - Roche Pharma Research and Early Development, Roche Innovation Center Basel, Basel, Switzerland

For peer review only

For peer review only - http://bmjopen.bmj.com/site/about/guidelines.xhtml

41 - South London and Maudsley NHS Foundation Trust (SLaM), London SE5 8AZ, UK

42 - Institute for Translational Neurodevelopment, Institute of Psychiatry, Psychology & Neuroscience, King's College London, London WC2R 2LS, UK

43 - SickKids Research Institute, Peter Gilgan Centre for Research and Learning, Hospital for Sick Children, Toronto, Canada.

44 - Child and Youth Division Centre for Addiction and Mental Health, CAMH

45 - Department of Psychiatry, Temerty Faculty of Medicine, University of Toronto

46 - F. Hoffman-La Roche Ltd, Basel, Switzerland

Corresponding author

Dr Alexandra Lautarescu, Department of Psychology, Institute of Psychiatry, Psychology & Neuroscience, King's College London,

Alexandra.Lautarescu@kcl.ac.uk

For peer review only - http://bmjopen.bmj.com/site/about/guidelines.xhtml

ABSTRACT

Introduction: Autism is a common neurodevelopmental condition with a complex genetic aetiology that includes contributions from monogenic and polygenic factors. Many autistic people have unmet healthcare needs that could be served by genomics-informed research and clinical trials. The primary aim of the European Autism GEnomics Registry (EAGER) is to establish a registry of participants with a diagnosis of autism or an associated rare genetic condition who have undergone whole-genome sequencing. The registry can facilitate recruitment for future clinical trials and research studies, based on genetic, clinical, and phenotypic profiles, as well as participant preferences. The secondary aim of EAGER is to investigate the association between mental and physical health characteristics and participants' genetic profiles.

Methods and analysis*:* EAGER is a European multisite cohort study and registry and is part of the AIMS-2-TRIALS consortium. EAGER was developed with input from the AIMS-2-TRIALS Autism Representatives and representatives from the rare genetic conditions community. 1,500 participants with a diagnosis of autism or an associated rare genetic condition will be recruited at 13 sites across 8 countries. Participants will give a blood or saliva sample for whole-genome sequencing and answer a series of online questionnaires. Participants may also consent for the study to access pre-existing clinical data. Participants will be added to the EAGER registry and data will be shared externally through established AIMS-2-TRIALS mechanisms.

Ethics and dissemination: To date, EAGER has received full ethical approval for 11 out of the 13 sites in the UK (REC 23/SC/0022), Germany (S-375/2023), Portugal (CE-085/2023), Spain (HCB/2023/0038, PIC-164-22), Sweden (Dnr 2023-06737-01),

For peer review only - http://bmjopen.bmj.com/site/about/guidelines.xhtml

Ireland (230907) and Italy (CET_62/2023, CEL-IRCCS OASI/24-01-2024/EM01, EM 2024-13/1032 EAGER). Findings will be disseminated via scientific publications and conferences, but also beyond to participants and the wider community (e.g., the AIMS-2-TRIALS website, stakeholder meetings, newsletters).

STRENGHTS AND LIMITATIONS OF THIS STUDY

- Data from full genotyping through whole-genome sequencing will be combined with mental and physical health data and participant research priorities
- The EAGER sample (n=1,500), although relatively small for genetic analyses, will include a substantial proportion (around one third) of participants with a rare genetic condition, ensuring that heterogeneous presentations across the autism spectrum are captured
- The EAGER registry will improve the speed, efficiency, and impact of research studies and clinical trials across Europe with a culturally diverse cohort of re-contactable participants, and shared data
- EAGER was developed with input from the AIMS-2-TRIALS Autism Representatives and representatives from the rare genetic conditions community
- Phenotypic data are collected only via self/informant-report questionnaires and not direct clinical assessments

1. INTRODUCTION

Autism is a neurodevelopmental condition diagnosed on the basis of differences in social communication and interaction, sensory processing, restricted and repetitive behaviours, and intense interests (1,2), with a prevalence of around 1-2% of the general population (3). Autism is associated with a very large number of common

For peer review only - http://bmjopen.bmj.com/site/about/guidelines.xhtml

genetic variants of small effect (4), as well as with several rare genetic conditions (e.g., Dup15q syndrome, Rett syndrome, tuberous sclerosis complex, Angelman syndrome) where autism prevalence is as high as 30-93% (5,6).

Many autistic people and people with associated rare genetic conditions have co-occurring mental and physical health conditions that impact quality of life. For example, depression, anxiety, gastrointestinal symptoms, and epilepsy commonly co-occur with autism (7–12). For some autistic people and those with associated rare genetic conditions, many of these conditions can be severe and life-limiting, including respiratory and cardiac disorders in Rett syndrome (13,14), hyperphagia in Prader-Willi syndrome (15), and tumour growth in tuberous sclerosis complex (16). This makes the development of treatments and support for these populations a crucial area of research. Further, some autistic people may find aspects of being autistic distressing and disabling and may want help with these (17–20). All autistic people have the right to evidence-based care and support for aspects that are detrimental to their quality of life and wellbeing (21). Despite this, there is currently a paucity of personalised evidence-based care and support for autistic people across the spectrum (22,23), and polypharmacy (i.e., taking multiple medications) for various co-occurring symptoms is commonplace (24).

With a heritability of around 80%, autism has strong genetic influences (e.g., 25,26). However, to date, there is no known gene that, when mutated, increases the likelihood of an autism diagnosis without also increasing the likelihood of intellectual disability or other neurodevelopmental conditions (27). Consequently, genetic

For peer review only

For peer review only - http://bmjopen.bmj.com/site/about/guidelines.xhtml

research is an important biomedical tool in the study of autism and associated rare genetic conditions, with benefits including personalised medicine, early identification of health risks, a better self-understanding, and a sense of community (28). To aid the development of evidence-based care for these populations, it is crucial to better understand genetic differences between syndromic forms of autism and autism without a known highly penetrant genetic influence. Further, by linking genetic mechanisms to treatment outcomes, accurate information can be offered regarding potential difficulties, as well as regarding interventions that may be effective for aspects of their lived experience that they want or need support with.

Using extensive genetic and phenotypic data, the European Autism GEnomics Registry (EAGER) will enable a better understanding of the genetic architecture of autism and associated rare genetic conditions, as well as the relationship between genetics and outcomes such as health, quality of life, and wellbeing. The EAGER registry will also allow participants to be recontacted for future research studies or clinical trials that may be suitable for them. This will facilitate research by streamlining and accelerating recruitment across Europe, as well as bring together key research and clinical institutions, to the benefit of participants and their families. In line with tailoring evidence-based care to specific populations, clinical trials that are genetically-informed allow researchers to invite participants who are the most suited to, and may benefit most from, a treatment (29). Selective recruitment at the early stages of treatment development encourages the downstream development of personalised medicine.

This project aims to be a catalyst for future research and clinical trials in autism and associated rare genetic conditions in Europe. This will be achieved by connecting

For peer review only

For peer review only - http://bmjopen.bmj.com/site/about/guidelines.xhtml

key sites and allowing research groups and pharmaceutical companies planning research studies or clinical trials to have access to a European registry of people who have consented to being recontacted. EAGER data will be shared via established AIMS-2-TRIALS data sharing mechanisms via platforms such as ELIXIR (https://elixir-europe.org/) and the Autism Sharing Initiative (ASI). The ASI is a global collaboration connecting autism data through federated mechanisms (30). The genetic, phenotypic, and clinical data collected in EAGER will be analysed with other international autism datasets such as MSSNG (31), POND (32), LEAP (33), SPARK (34), and Searchlight (35), providing the opportunity to investigate key research questions.

2. METHODS AND ANALYSIS

2.1. Study design and population

EAGER is a European multisite study that aims to create a registry of 1,500 adults and children with a diagnosis of autism or a rare genetic condition associated with autism. Recruitment for EAGER will take place at 13 sites across 8 European countries (see figure 1), with the recruitment and data collection protocol being standardised across all sites.

Figure 1. EAGER collaborating centres

To be eligible, participants need to be over 2 years of age and have a diagnosed rare genetic condition associated with autism and/or have a diagnosis of autism (see figure 2 for examples of conditions). Decisions regarding which conditions and genetic variants to prioritise have been made on the basis of published literature (36)

For peer review only - http://bmjopen.bmj.com/site/about/guidelines.xhtml

and discussions with experts in the field (TB, LG), with consideration given to the penetrance, prevalence, and association with autism. In addition, given the difficulty of recruiting cohorts of meaningful sizes for each of these conditions, pragmatic aspects related to participant access at each of the EAGER sites were also taken into consideration. For participants with a diagnosed rare genetic condition, recruitment will include conditions that are associated with autism or autism-like phenotypes, such as those listed in figure 2. Given that autism assessments are not routinely performed for all people with rare genetic conditions, an autism diagnosis is not required for inclusion into the study for participants in this group. For participants with a diagnosis of autism only, and where existing genetic information is available, recruitment will prioritise individuals within multiplex families and individuals with genetic variants associated with autism (see figure 2). Participants will not be excluded from the study based on any other characteristics such as co-occurring conditions, sex, or intellectual disability.

Figure 2. EAGER protocol summary

2.2. Patient and public involvement and ethical consultations

Genetic research can involve complex ethical challenges, making it crucial to prioritise ethical considerations and participatory research practices (21,28). Participatory research practices can help ensure that the interests of our participants are appropriately represented and that the study is beneficial to its participants and the wider community. EAGER was developed with crucial input and feedback from community representatives via dedicated working groups. Our autism working group consists of AIMS-2-TRIALS Autism Representatives: autistic people and family

For peer review only

For peer review only - http://bmjopen.bmj.com/site/about/guidelines.xhtml

members or carers of autistic people. Our rare genetic conditions working group consists of parents/carers of people with rare genetic conditions and/or representatives from European rare genetic conditions associations. As EAGER was conceptualised and funded prior to the establishment of our lived experience working groups, it is not a fully participatory research endeavour. This was discussed with and understood by all members of the working groups at the outset of our collaboration. Within these constraints, every effort was made to consult with our lived experience partners throughout the lifespan of this project, and feedback was integrated to the greatest degree possible.

The EAGER working groups have offered invaluable advice and feedback to guide the planning process of EAGER (see Box 1), either as part of working group meetings or through communication via email or contributions to collaborative documents. Open discussion within these groups was encouraged; as the groups included a variety of perspectives, there were naturally different solutions to questions. When no consensus was reached, the core research team made the final decision based on feasibility.

Box 1. Key examples of how patient and public involvement guided the EAGER study

- Refining the study rationale and clarifying perceived benefit of the study for autistic people
- Deciding on study name and logo
- Discussing ethical implications of the research, ensuring that appropriate safeguards are in place and that any limitations are transparently communicated to participants
- Ensuring that the information presented in study documents is clear, accessible, transparent,

For peer review only - http://bmjopen.bmj.com/site/about/guidelines.xhtml

and sufficient to allow participants to make an informed decision about their participation

- Creating a "Data sharing Frequently Asked Questions" document to accompany the information sheet
- Developing different versions of information sheets tailored to the needs of our different participant groups (e.g., easy-read, adolescent information sheet)
- Ensuring that the consent forms are clear and transparent about what precisely a participant is consenting to
- Ensuring that the language used in study documents is appropriate and in line with community preferences
- Discussing the phenotypic measures used in the study and suggesting changes to the content/language of questionnaires
- Sharing all key study documents for feedback prior to submission for ethical approval
- Clearly stating that EAGER is against eugenics / prenatal testing for autism on participant-facing documents
- Detailing exactly what analyses will be performed on participant data and making it clear that no further analyses will occur without reconsenting
- Clarifying that analyses performed on EAGER data will need to be approved by a committee before they can begin
- Clarifying in the ethics documents that not all decisions were made based on consensus
- Adding a disclaimer in the protocol/ethics with regards to the fact that citing papers does not represent an endorsement of the language/aims of those papers or of the authors

Further, we conducted consultation with other stakeholders, ethical advisors, and additional working groups within the consortium, in order to evaluate risks and potentially sensitive implications of this research. We hope that the aforementioned groups will guide EAGER in the most beneficial direction for its participants and the wider community. These groups will continue to be consulted throughout the lifetime of the project.

For peer review only - http://bmjopen.bmj.com/site/about/guidelines.xhtml

2.3. Participant recruitment, information sheets, and informed consent

Potential participants will be approached by representatives at each local site (i.e., members of the clinical or research team). Each site will screen and recruit participants based upon recontact of participants from prior research studies or prior/current patients that fit the inclusion criteria. Recruitment targets have been allocated to each of the sites based on local capacity and cohort availability, with targets ranging from n=50 to n=120 per site. The distribution of participant types between sites also differs based on capacity, with some sites recruiting a majority of autistic participants (e.g., 90% of target sample) and other sites recruiting a majority of participants with rare genetic conditions (e.g., 70% of target sample).

Written informed consent will be required for participation in the study and participants will be provided with information sheets tailored to their age and levels of intellectual disability (see figure 2 for details on documents for specific participant groups). A personal consultee will be asked to consent on behalf of individuals who are deemed to not have capacity to consent following a capacity to consent assessment. Due to the high prevalence of co-occurring intellectual disability in people with rare genetic conditions, these may make up a significant proportion of the overall sample. Parental consent will be required for those under the legal age of consent (e.g., 16 years old in the UK).

For peer review only - http://bmjopen.bmj.com/site/about/guidelines.xhtml

To take part in EAGER, participants must consent to core aspects of the study: giving a blood or saliva sample (or permission to use a pre-existing sample), completing questionnaires, being added to the registry, and data sharing.

2.4. Biological sample collection

Participants will give a blood or saliva sample, depending on preference and appropriateness. For blood samples, peripheral venous blood will be collected in ethylenediaminetetraacetic acid (EDTA)-coated vacucontainers at each local site by experienced phlebotomists or other professionals who are trained in collecting blood samples. For saliva collection, the Oragene®•DNA self-collection kits, as well as assisted collection kits for those unable to collect their own sample, will be used. If existing blood or saliva samples are available and have been stored appropriately, they can be used in lieu of a new sample, with participant consent.

2.5. Genetic analysis

2.5.1. DNA Quality control

All concentrations will be measured in fluorescence (Quant-IT kit), in duplicate; samples with low concentrations will be quantified with a more sensitive kit. Samples with initial concentrations higher than 120 ng/μL will be diluted to concentrations compatible with the working conditions of the platform and samples with heterogeneous results in the first measurements will be verified by a second series of measurements. The quality of the samples will be evaluated on all samples by migrating a small amount of DNA on TapeStation Agilent 4200, as well as a polymerase chain reaction (PCR) amplification test (simultaneous amplification of two microsatellite markers) and a verification of the sex of individuals by PCR. With

For peer review only - http://bmjopen.bmj.com/site/about/guidelines.xhtml

few exceptions, samples with a DNA Integrity Number < 6 or showing a sex mismatch with the file information will be excluded.

2.5.2. Whole genome sequencing

After quality control, the preparation of the libraries will be performed on the automated platform of the National Center for Genomic Research (CNRGH). Fragmentation of the genomic DNA with ultrasound (Covaris), will use the protocol of the Illumina TruSeq DNA PCR-free kit with a starting quantity of 1 ug of genomic DNA. Quality control will be systematically performed after preparation of the libraries by electropherogram verification (Bioanalyzer 3 profile) and quantitative PCR (Light cycler 480, Roche). Clustering capacity will be assessed by sequencing 1 million reads on Miseq. Sequencing will be performed in pair-end (2x150 bases) on the Illumina NovaSeq X platform of the CNRGH, using the corresponding sequencing kits and according to the supplier's specifications. Quality control will be performed during sequencing (intensity, Q30, phasing/prephasing/base proportion). A minimum quantity of sequences allowing an average depth of about 30x for each sample will be generated.

2.5.3. Variant calling SNVs, Indels, and CNVs

After aligning the genomes to the GRCh38 human reference, variants will be called jointly for the cohort using DeepVariant 1.6.0 and GLnexus 1.4.1. All multiallelic variants will be decomposed and indels will be normalized using bcftools 1.15 with the norm mode. Functional annotation will be performed using VEP Ensembl 111. Copy number variants (CNVs) will be identified using ClinSV v1.0.1, which combines Lumpy and CNVnator to look for discordantly mapping read pairs, split-mapping

For peer review only - http://bmjopen.bmj.com/site/about/guidelines.xhtml

reads and depth of coverage changes. A table with rare gene affecting variants will be generated containing all variants passing the variant detection threshold (PASS and HIGH) and having a population allele frequency ≤1%.

2.5.4. Variant validation

Validation on Compressed Reference-oriented Alignment Maps (CRAMS) will use Integrative Genomics Viewer (IGV): for single nucleotide variants (SNVs) and Indels, after building a list of candidate *de novo* variants using slivar 0.2.8, we will exclude those located in Low Complexity Regions. We will then use a machine learning approach to predict for each of those candidates *de novo* variants whether they were true or false positives. This approach will be calibrated using a learning dataset of ~900 variants manually validated from independent cohorts sequenced using the same methodology, allowing a false positive rate of ≤ 5%. We will then validate the CRAM files visually using IGV for all the *de novo* variants in genes related to neurodevelopmental conditions. Sanger sequencing will be used to test *de novo* loss-of-function or missense with Missense badness, PolyPhen-2, and Constraint scores ≥ 2.

2.5.5. Ancestry

Principal components analysis (PCA) of the variance standardized relationship matrix will be used to evaluate the ancestry of individuals in the EAGER cohort and to provide components for any covariate adjustments. To find subpopulation clusters, we will compute the admixture (admixture 1.3.0) of the cohort along with a reference population (*e.g.* 1000 genomes, Simons Genome Diversity Project), and then apply a machine learning approach to predict ancestries.

For peer review only - http://bmjopen.bmj.com/site/about/guidelines.xhtml

2.5.6. Polygenic scores

Each participant will have a polygenic profile on common variants computed from WGS. Polygenic scores will be computed using state-of-the-art approaches such as SBayesR (GCTB 1.05beta) or SBayesRC 0.2.4. Each polygenic score will be standardised on neurotypical individuals according to their ancestry.

2.5.7. Genetic profile of participants

Participants will have a genetic profile that includes rare and common variants associated with neurodevelopmental conditions. Several databases (SFARI/SPARK, DBD, SysNDD, DDG2P) have been developed to provide extensive information on these genes. An updated list of more than 1,800 genes associated with neurodevelopmental conditions (i.e., High-Confidence genes) can be downloaded on the Gene Trek website. These are genes with strong evidence for genetic association with neurodevelopmental conditions (i.e., SFARI "category 1" genes, DBD "Tier 1" or "autosomal recessive" inheritance list, DDG2P "definitive" confidence category and "Brain/Cognition" organ specificity, SysNDD "Definitive" category, and all SPARK genes). For common variants through the polygenic scores, a list of summary statistics for traits in different domains of interest will be built (e.g., psychiatric traits such as anxiety, neurodevelopmental conditions such as autism or personal traits such as subjective well-being).

2.6. Measures

Following collection of the biological sample, participants will be given access to an online form hosted on the Qualtrics platform, with a series of questionnaires,

For peer review only - http://bmjopen.bmj.com/site/about/guidelines.xhtml

focussing on autism, rare genetic conditions, co-occurring physical and mental health conditions, opinions on research priorities, and quality of life and wellbeing (see table 1). These will be a mixture of validated and non-validated questionnaires that have been adapted for EAGER, and have been chosen to overlap with other major initiatives or studies both within AIMS-2-TRIALS (37,38) and externally, such as MSSNG (31), POND (32), LEAP (33), SPARK (34), and Simon's Searchlight (35). All questionnaires have been translated into the local language of each site from English. The online questionnaires will be completed by participants answering on behalf of themselves, or by parents and consultees answering on behalf of the participant.

Table 1. EAGER questionnaires

Domain	Measure	Description	Respondents
**Autism traits**	Social Responsiveness Scale (SRS (39))	65 items: Autism traits (including social awareness, cognition, communication, motivation, and repetitive interests and behaviours)	Adult self-report
	Social Communication Questionnaire (SCQ, (40))	40 items: Autism traits (including communication skills and social functioning)	Parent or consultee report for child or adult
**Mental health**	Strengths and Difficulties Questionnaire (SDQ, (41))	25 items: General mental health across five scales (conduct problems, hyperactivity, emotional problems, peer problems, prosocial behaviour)	Parent or consultee report for adult or child
	Patient Health Questionnaire-9 (PHQ-9, (42))	9 items: Core features of major depressive disorder	Adult self-report

For peer review only - http://bmjopen.bmj.com/site/about/guidelines.xhtml

	Patient Health Questionnaire-A (PHQ-A, (43))	9 items: Core features of major depressive disorder*	Adolescent (16 and 17 years) self-report
	Generalised Anxiety Disorder-7 (GAD-7, (44))	7 items: Core features of generalised anxiety disorder	Adult self-report
**Quality of life**	World Health Organization Quality of Life-BREF (WHO-QOL-BREF, (45))	26 items: Quality of life across four domains (physical health, psychological health, social relationships, environment)	Adult self-report
	Cantrill Ladder (46)	1 item: Subjective quality of life (10 point scale from "worst possible life" to "best possible life")	Adult self-report. Parent or consultee report for child or adult.
**Mental wellbeing**	Warwick-Edinburgh Mental Wellbeing Scales (WEMWBS, (47))	14 items: Mental wellbeing and health	Adult self-report. Parent or consultee self-report+
**Restrictive and repetitive behaviours**	Adult Routines inventory (ARI, (48))	55 items: Restrictive and repetitive behaviours	Adult self-report
	Repetitive Behaviours Scale-Revised (RBS-R, (49))	43: Assessing restrictive and repetitive behaviours	Parent or consultee report for child or adult
**ADHD**	Conners' Adult ADHD Rating Scales (CAARS, (50))	26 items: Core features of ADHD	Adult self-report
	ADHD Rating Scale (51))	18 items: Core features of ADHD	Parent report for child Consultee report for adult^
**Demographics**	Non-validated questionnaire	16-25 items: Demographic characteristics	Adult self-report. Parent or consultee report for child or adult.

For peer review only - http://bmjopen.bmj.com/site/about/guidelines.xhtml

**Co-occurring conditions**	Non-validated questionnaire (adapted from Simons Searchlight (35))	Items assessing 168 diagnosed and self-diagnosed psychiatric, neurodevelopmental, and physical health conditions.	Adult self-report. Parent or consultee report for child or adult.
**Medications**	Non-validated questionnaire	1 item: Number of medications taken by participant per day	Adult self-report. Parent or consultee report for child or adult.
**General functioning**	Non-validated questionnaire (adapted from (52))	7-11 items: Seizures, gross motor skills, language and communication, intellectual disability, support needs, sleep	Adult self-report. Parent or consultee report for child or adult.
**Voice of the community**	Non-validated questionnaire (adapted from Simon Searchlight (35))	7-13 items: Priorities for research	Adult self-report. Parent or consultee report for child or adult.

* Additional questions and assessment of suicidality in the original measure were removed for the purpose of this study and only the first core 9 items were included.

^ The ADHD rating scale was adapted for use in EAGER to make it appropriate for adults and remove child-specific language.

+ Administered to parents and caregivers in relation to their own mental wellbeing.

2.7. Data analysis

EAGER will collect genetic and phenotypic data, which can be analysed both alone and in conjunction with other data collected within AIMS-2-TRIALS, as well as with shared data from other cohorts (e.g., via ELIXIR or the ASI). Research questions will be decided in collaboration with our community working groups, but a few brief examples are outlined below.

Using the genetic and phenotypic data, we can answer questions such as:

For peer review only - http://bmjopen.bmj.com/site/about/guidelines.xhtml

- Identifying genetic variants that are associated with an autism diagnosis and/or autism phenotypic characteristics (e.g., scores on SRS and SCQ)
- Identifying genetic variants that are associated with co-occurring conditions such as neurodevelopmental (e.g., scores on CAARS and ADHD rating scale) or mental health conditions (e.g., scores on SDQ, PHQ-9, and GAD-7)
- Identifying genetic variants that are associated with characteristics such as quality of life (e.g., Cantrill ladder, WHO-QOL-BREF) or functioning (e.g., general functioning questionnaire).

Using the phenotypic data, we can answer questions such as:

- Identifying participants' priorities for future research (e.g., Voice of the Community survey) and assessing the factors that may influence responses (e.g., diagnoses, age).
- Identifying associations between participant diagnoses (including co-occurring conditions) and subjective experiences such as quality of life (e.g., Cantrill ladder, WHO-QOL-BREF) or mental wellbeing (e.g., WEMWBS)

The sample size of this study (n=1,500), which was driven by the available budget, is moderate-to-large in the field and will be significantly enhanced via the planned data sharing and integration. While EAGER will not recruit a control group, we expect there to be significant opportunities for analyses where participant subgroups can be identified and compared (e.g., participants with epilepsy compared to participants without epilepsy). The deep phenotyping and the expected heterogeneity within the sample will enable us to better understand differences between autistic people with and without a known genetic cause.

3. DATA SHARING AND EAGER REGISTRY

For peer review only - http://bmjopen.bmj.com/site/about/guidelines.xhtml

3.1. Data sharing

Currently, funding bodies and participant groups have identified the need for research groups to share their data to maximise the value of each research contribution, pool data to address research questions that require a larger number of participants, or to carry out meta-analyses (53). For this reason, EAGER data will be shared with the autism research community. Data from AIMS-2-TRIALS studies (including EAGER) are stored centrally on the OWEY platform developed by and hosted at Institut Pasteur in Paris, France. This enables researchers within the AIMS-2-TRIALS consortium to access and use the data. External data sharing is done via the ELIXIR platform, in accordance with the AIMS-2-TRIALS data sharing policy. In addition, EAGER data will be shared via the ASI platform, as detailed below. With participant consent, EAGER data (genetic data, data from online questionnaires, and existing data held at local sites) will be shared with any explicit identifiers removed. Rigorous safeguards will be implemented for access to data via established AIMS-2-TRIALS mechanisms and via the ASI platform (30). Regardless of the data sharing mechanism, AIMS-2-TRIALS researchers will act as data custodians and retain control over who the data are shared with and accessed by. Researchers will not be able to access EAGER data without approval from a data access committee, which will consist of autistic and non-autistic people, researchers, and members of the rare genetic community.

3.1.1. Autism Sharing Initiative (ASI)

The ASI is a new collaboration working to create the first federated, global network for sharing genomic and clinical data in autism research. The ASI is led by software

For peer review only - http://bmjopen.bmj.com/site/about/guidelines.xhtml

company DNAStack and involves other collaborators such as Autism Speaks, Roche, and the Ontario Brain Institute (please see (54) for the full list). This is an ongoing project and new collaborators may be added.

Data sharing within the ASI will be built upon decentralised, federated data sharing principles. Data federation allows researchers to search, access, and analyse data that reside in different locations and are connected via a network, without the need for data to be moved between institutions/researchers. Data federation allows researchers to interrogate and analyse data hosted in different locations, in compliance with data regulations and improving privacy and security. A federated data network involves connecting individual datasets, which remain in their protected local environments (i.e., at the research institution where the data was collected), across one cloud-based network. To our knowledge, the ASI carries no additional risk to participant re-identification than traditional data sharing. The ASI software is built upon security standards developed by the Global Alliance for Genomics and Health (55), and regularly updated in order to ensure that data are safeguarded. Within the ASI, data will include genomic, multi-omic, medical, and clinical data from international autism research sources.

3.2. EAGER registry

The EAGER registry will allow participants to be contacted for future clinical trials and research studies. Researchers viewing shared participant data (i.e., regardless of data sharing mechanism) will be able to contact the coordinating site (King's College London) to request specific participant groups based upon their desired criteria without access to participant personal information. King's College London will

For peer review only - http://bmjopen.bmj.com/site/about/guidelines.xhtml

pass these requests onto the relevant local sites, who can send information about the study or clinical trial to participants. AIMS-2-TRIALS is funded until May 2025. Sustainability is a core aspect of the work done as part of the consortium and plans are in place to ensure that EAGER will continue to be supported through future funding initiatives currently being pursued by the investigators.

4. ETHICS AND DISSEMINATION

4.1. Ethics

EAGER has received full ethical approval for 11 out of the 13 sites, with approval pending from an ethics committee in France and one in Italy. Approval has been received for all sites in the UK (REC 23/SC/0022), Germany (S-375/2023), Portugal (CE-085/2023), Spain (HCB/2023/0038, PIC-164-22), Sweden (Dnr 2023-06737-01) and Ireland (230907) and three sites in Italy (CET_62/2023, CEL-IRCCS OASI/24-01-2024/EM01, EM 2024-13/1032 EAGER). The protocol described here is being followed by each of the sites and is based on the approved UK documents. Please refer to the above section "Patient and Public Involvement and Ethics Consultation" for detail on ethics and discussions within our community working groups.

4.2. Dissemination

We plan to disseminate our findings widely via academic avenues, such as publications in peer-reviewed major international scientific journals and conferences. In addition, we will disseminate beyond academic avenues to ensure that our findings reach participants and the wider communities, including the AIMS-2-TRIALS

For peer review only - http://bmjopen.bmj.com/site/about/guidelines.xhtml

website (www.aims-2-trials.eu), stakeholder meetings, and newsletters. As part of the overall AIMS-2-TRIALS project, we have access to a European network including researchers, clinicians, and families within the autism community. This network will also provide a forum for dissemination of research findings and training opportunities.

Disclaimer: Citing papers does not represent an endorsement of the language used in these papers, nor of the aims of the research or researchers.

REFERENCES

1. Lord C, Brugha TS, Charman T, Cusack J, Dumas G, Frazier T, et al. Autism spectrum disorder. Nat Rev Dis Primers. 2020 Jan 16;6(1):1–23.

2. Kodak T, Bergmann S. Autism Spectrum Disorder: Characteristics, Associated Behaviors, and Early Intervention. Pediatr Clin North Am. 2020 Jun;67(3):525–35.

3. Zeidan J, Fombonne E, Scorah J, Ibrahim A, Durkin MS, Saxena S, et al. Global prevalence of autism: A systematic review update. Autism Res. 2022 May;15(5):778–90.

4. Willsey HR, Willsey AJ, Wang B, State MW. Genomics, convergent neuroscience and progress in understanding autism spectrum disorder. Nat Rev Neurosci. 2022 Jun;23(6):323–41.

5. Richards C, Jones C, Groves L, Moss J, Oliver C. Prevalence of autism spectrum disorder phenomenology in genetic disorders: a systematic review and meta-analysis. The Lancet Psychiatry. 2015 Oct 1;2(10):909–16.

For peer review only

For peer review only - http://bmjopen.bmj.com/site/about/guidelines.xhtml

6. Lusk L, Vogel-Farley V, DiStefano C, Jeste S. Maternal 15q Duplication Syndrome. In: Adam MP, Ardinger HH, Pagon RA, Wallace SE, Bean LJ, Gripp KW, et al., editors. GeneReviews® [Internet]. Seattle (WA): University of Washington, Seattle; 1993 [cited 2022 May 9]. Available from: http://www.ncbi.nlm.nih.gov/books/NBK367946/

7. Lai MC, Kassee C, Besney R, Bonato S, Hull L, Mandy W, et al. Prevalence of co-occurring mental health diagnoses in the autism population: a systematic review and meta-analysis. Lancet Psychiatry. 2019 Oct;6(10):819–29.

8. Tye C, Runicles AK, Whitehouse AJO, Alvares GA. Characterizing the Interplay Between Autism Spectrum Disorder and Comorbid Medical Conditions: An Integrative Review. Front Psychiatry. 2018;9:751.

9. van Heijst BF, Deserno MK, Rhebergen D, Geurts HM. Autism and depression are connected: A report of two complimentary network studies. Autism. 2020 Apr;24(3):680–92.

10. Hollocks MJ, Lerh JW, Magiati I, Meiser-Stedman R, Brugha TS. Anxiety and depression in adults with autism spectrum disorder: a systematic review and meta-analysis. Psychological Medicine. 2019 Mar;49(4):559–72.

11. Wang J, Ma B, Wang J, Zhang Z, Chen O. Global prevalence of autism spectrum disorder and its gastrointestinal symptoms: A systematic review and meta-analysis. Front Psychiatry. 2022 Aug 23;13:963102.

12. Besag FM. Epilepsy in patients with autism: links, risks and treatment challenges. Neuropsychiatr Dis Treat. 2017 Dec 18;14:1–10.

For peer review only

593 13. Acampa M, Guideri F. Cardiac disease and Rett syndrome. Arch Dis Child. 2006 May;91(5):440–3.

595 14. Katz DM, Dutschmann M, Ramirez JM, Hilaire G. Breathing Disorders in Rett Syndrome: Progressive Neurochemical Dysfunction in the Respiratory Network after Birth. Respir Physiol Neurobiol. 2009 Aug 31;168(1–2):101–8.

598 15. Driscoll DJ, Miller JL, Cassidy SB. Prader-Willi Syndrome. In: Adam MP, Mirzaa GM, Pagon RA, Wallace SE, Bean LJ, Gripp KW, et al., editors. GeneReviews® [Internet]. Seattle (WA): University of Washington, Seattle; 1993 [cited 2023 Sep 18]. Available from: http://www.ncbi.nlm.nih.gov/books/NBK1330/

602 16. Islam MP. Tuberous Sclerosis Complex. Semin Pediatr Neurol. 2021 Apr;37:100875.

604 17. Schaaf RC, Toth-Cohen S, Johnson SL, Outten G, Benevides TW. The everyday routines of families of children with autism: examining the impact of sensory processing difficulties on the family. Autism. 2011 May;15(3):373–89.

607 18. Green D, Chandler S, Charman T, Simonoff E, Baird G. Brief Report: DSM-5 Sensory Behaviours in Children With and Without an Autism Spectrum Disorder. J Autism Dev Disord. 2016 Nov 1;46(11):3597–606.

610 19. Lai MC, Anagnostou E, Wiznitzer M, Allison C, Baron-Cohen S. Evidence-based support for autistic people across the lifespan: maximising potential, minimising barriers, and optimising the person-environment fit. Lancet Neurol. 2020 May;19(5):434–51.

For peer review only

614 20. Lord C, Charman T, Havdahl A, Carbone P, Anagnostou E, Boyd B, et al. The Lancet Commission on the future of care and clinical research in autism. The Lancet. 2022 Jan;399(10321):271–334.

617 21. Heraty S, Lautarescu A, Belton D, Boyle A, Cirrincione P, Doherty M, et al. Bridge-building between communities: Imagining the future of biomedical autism research. Cell. 2023 Aug 31;186(18):3747–52.

620 22. Sandbank M, Bottema-Beutel K, Crowley S, Cassidy M, Dunham K, Feldman JI, et al. Project AIM: Autism intervention meta-analysis for studies of young children. Psychological Bulletin. 2020;146(1):1–29.

623 23. Braga LAM, Conte Filho CG, Mota FB. Future of genetic therapies for rare genetic diseases: what to expect for the next 15 years? Ther Adv Rare Dis. 2022 Jun 10;3:26330040221100840.

626 24. Feroe AG, Uppal N, Gutiérrez-Sacristán A, Mousavi S, Greenspun P, Surati R, et al. Medication Use in the Management of Comorbidities Among Individuals With Autism Spectrum Disorder From a Large Nationwide Insurance Database. JAMA Pediatrics. 2021 Sep 1;175(9):957–65.

630 25. Bai D, Yip BHK, Windham GC, Sourander A, Francis R, Yoffe R, et al. Association of Genetic and Environmental Factors With Autism in a 5-Country Cohort. JAMA Psychiatry. 2019 Oct 1;76(10):1035–43.

633 26. Tick B, Bolton P, Happé F, Rutter M, Rijsdijk F. Heritability of autism spectrum disorders: a meta-analysis of twin studies. Journal of Child Psychology and Psychiatry. 2016;57(5):585–95.

For peer review only

27. Myers SM, Challman TD, Bernier R, Bourgeron T, Chung WK, Constantino JN, et al. Insufficient Evidence for “Autism-Specific” Genes. Am J Hum Genet. 2020 May 7;106(5):587–95.

28. Natri HM, Chapman CR, Heraty S, Dwyer P, Walker N, Kapp SK, et al. Ethical challenges in autism genomics: Recommendations for researchers. European Journal of Medical Genetics. 2023 Sep 1;66(9):104810.

29. Díaz-Caneja CM, State MW, Hagerman RJ, Jacquemont S, Marín O, Bagni C, et al. A white paper on a neurodevelopmental framework for drug discovery in autism and other neurodevelopmental disorders. Eur Neuropsychopharmacol. 2021 Jul;48:49–88.

30. DNAstack [Internet]. [cited 2023 Oct 8]. DNAstack launches Neuroscience AI to accelerate collaborative research and discovery, DNAstack. Available from: https://dnastack.com/news-neuroscience-ai-launch-announcement/

31. MSSNG [Internet]. [cited 2023 Sep 18]. Available from: https://research.mss.ng/

32. POND. Your Research Priorities [Internet]. Province of Ontario Neurodevelopmental Network. [cited 2023 Apr 11]. Available from: https://pond-network.ca/your-research-priorities/

33. Charman T, Loth E, Tillmann J, Crawley D, Wooldridge C, Goyard D, et al. The EU-AIMS Longitudinal European Autism Project (LEAP): clinical characterisation. Molecular Autism. 2017 Jun 23;8(1):27.

34. SPARK [Internet]. [cited 2023 Sep 18]. Available from: https://sparkforautism.org/

For peer review only

35. SFARI [Internet]. 2019 [cited 2023 Sep 18]. SFARI | Simons Searchlight. Available from: https://www.sfari.org/resource/simons-searchlight/

36. Rolland T, Cliquet F, Anney RJL, Moreau C, Traut N, Mathieu A, et al. Phenotypic effects of genetic variants associated with autism. Nat Med. 2023 Jul;29(7):1671–80.

37. AIMS-2-TRIALS - Autism Research For Europe [Internet]. 2018 [cited 2023 Oct 8]. Available from: https://www.aims-2-trials.eu/

38. Oakley BFM, Loth E, Jones EJH, Chatham CH, Murphy DG. Advances in the identification and validation of autism biomarkers. Nat Rev Drug Discov. 2022 Oct;21(10):697–8.

39. Constantino JN, Davis SA, Todd RD, Schindler MK, Gross MM, Brophy SL, et al. Validation of a Brief Quantitative Measure of Autistic Traits: Comparison of the Social Responsiveness Scale with the Autism Diagnostic Interview-Revised. J Autism Dev Disord. 2003 Aug 1;33(4):427–33.

40. (SCQ) Social Communication Questionnaire [Internet]. [cited 2023 Sep 18]. Available from: https://www.wpspublish.com/scq-social-communication-questionnaire.html

41. Goodman R. The Strengths and Difficulties Questionnaire: a research note. J Child Psychol Psychiatry. 1997 Jul;38(5):581–6.

42. Kroenke K, Spitzer RL, Williams JB. The PHQ-9: validity of a brief depression severity measure. J Gen Intern Med. 2001 Sep;16(9):606–13.

For peer review only

For peer review only - http://bmjopen.bmj.com/site/about/guidelines.xhtml

43. Johnson JG, Harris ES, Spitzer RL, Williams JBW. The patient health questionnaire for adolescents: validation of an instrument for the assessment of mental disorders among adolescent primary care patients. J Adolesc Health. 2002 Mar;30(3):196–204.

44. Spitzer RL, Kroenke K, Williams JBW, Löwe B. A Brief Measure for Assessing Generalized Anxiety Disorder: The GAD-7. Archives of Internal Medicine. 2006 May 22;166(10):1092–7.

45. WHOQOL - Measuring Quality of Life| The World Health Organization [Internet]. [cited 2023 Sep 18]. Available from: https://www.who.int/tools/whoqol

46. Cantril H. The pattern of human concerns. New Brunswick, N.J.: Rutgers University Press; 1966. 427 p.

47. Tennant R, Hiller L, Fishwick R, Platt S, Joseph S, Weich S, et al. The Warwick-Edinburgh Mental Well-being Scale (WEMWBS): development and UK validation. Health and Quality of Life Outcomes. 2007 Nov 27;5(1):63.

48. Evans DW, Uljarević M, Lusk LG, Loth E, Frazier T. Development of Two Dimensional Measures of Restricted and Repetitive Behavior in Parents and Children. Journal of the American Academy of Child & Adolescent Psychiatry. 2017 Jan 1;56(1):51–8.

49. Bodfish JW, Symons FJ, Parker DE, Lewis MH. Repetitive Behavior Scale–Revised [Internet]. 2014 [cited 2023 Sep 18]. Available from: http://doi.apa.org/getdoi.cfm?doi=10.1037/t17338-000

For peer review only - http://bmjopen.bmj.com/site/about/guidelines.xhtml

50. Macey KD. Conners' Adult ADHD Rating Scales (CAARS). Archives of Clinical Neuropsychology. 2003 May 1;18(4):431–7.

51. DuPaul GJ, Power TJ, Anastopoulos AD, Reid R. ADHD Rating Scale—IV: Checklists, norms, and clinical interpretation. New York, NY, US: The Guilford Press; 1998. viii, 79 p. (ADHD Rating Scale—IV: Checklists, norms, and clinical interpretation).

52. Keute M, Miller MT, Krishnan ML, Sadhwani A, Chamberlain S, Thibert RL, et al. Angelman syndrome genotypes manifest varying degrees of clinical severity and developmental impairment. Mol Psychiatry. 2021 Jul;26(7):3625–33.

53. Lombardo MV, Lai MC, Baron-Cohen S. Big data approaches to decomposing heterogeneity across the autism spectrum. Mol Psychiatry. 2019 Oct;24(10):1435–50.

54. Digital Supercluster [Internet]. [cited 2023 Oct 8]. Autism Sharing Initiative. Available from: https://www.digitalsupercluster.ca/projects/autism-sharing-initiative/

55. Global Alliance for Genomics and Health. Enabling responsible genomic data sharing for the benefit of human health [Internet]. 2023 [cited 2023 Apr 11]. Available from: https://www.ga4gh.org/

For peer review only

AUTHOR CONTRIBUTIONS:

Conceptualisation: MB, AL, SH, SD, PV, RP, AG, KVdB, EE, CO, VR, JT, BO, DM, LG, TB, CC, TC. **Funding acquisition:** DM, CC, TC**. Project administration:** MB,

For peer review only - http://bmjopen.bmj.com/site/about/guidelines.xhtml

AL, DM, LG, TB, CC. TC. **Supervision:** AL, TC. **Visualisation:** MB, AL. **Data collection**: MB, AL, MA, RB, ABH, NB, SB, PB, JB, SC, RCE, MCB, JCF, PC, AD, RD, ME, MH, NM, GM, RM, CJM, SM, VM, APR, CPS, MS, KT, CT, FV, GO, BM, LG, TC. **Writing - original draft:** MB, AL, TC. **Writing - review and editing:** All authors

FUNDING STATEMENT:

This work has received funding from the Innovative Medicines Initiative 2 Joint Undertaking under grant agreement No 777394 for the project AIMS-2-TRIALS. This Joint Undertaking receives support from the European Union's Horizon 2020 research and innovation programme and EFPIA and AUTISM SPEAKS, Autistica, SFARI and from Horizon Europe [grant agreement no. 101057385] and from UK Research and Innovation (UKRI) under the UK government's Horizon Europe funding guarantee [grant no.10039383] (R2D2-MH). The funders had no role in the design of the study; in the collection, analyses, or interpretation of data; in the writing of the manuscript, or in the decision to publish the results. Any views expressed are those of the author(s) and not necessarily those of the funders. In addition, SB receives funding from Swedish Research Council and Region Stockholm. CT receives funding from Epilepsy Research UK, Autistica, the Baily Thomas Charitable Fund and the Tuberous Sclerosis Association. LG receives funding from Horizon Europe: Risk and Resilience in Developmental Diversity and Mental Health (R2D2-MH). CPS receives funding from Foundation of Prader-Willi Research, European Joint Program for Rare Diseases, Illumina, German Children's Cancer Foundation, and the German Ministry of Education and Research. RB, RM, SC, BM receive funding from the Italian Ministry of Health (Ricerca Corrente and 5x1000 to IRCCS

For peer review only - http://bmjopen.bmj.com/site/about/guidelines.xhtml

Fondazione Stella Maris). The EAGER study is funded by a Direct Financial Contribution by F Hoffmann La-Roche Ltd as part of the AIMS-2-TRIALS consortium funded by IMI/IHI. This paper represents independent research part-funded by the NIHR Maudsley Biomedical Research Centre at South London and Maudsley NHS Foundation Trust and King's College London. The views expressed are those of the author(s) and not necessarily those of the NIHR or the Department of Health and Social Care.

COMPETING INTEREST STATEMENT:

In the past 3 years TC has served as a paid consultant to F. Hoffmann-La Roche Ltd. and Servier and has received royalties from Sage Publications and Guilford Publications. DM has received funding for a PhD studentship from Compass, and for consulting from Jaguar Therapeutics and Hoffman Le Roche. GM receives funding for an investigator-initiated study from Compass Pathways; no financial or other conflict of interest with the present study. SB discloses that he has in the last 3 years acted as an author, consultant, or lecturer for Medice, Roche, and Linus Biotechnology. SB receives royalties for textbooks and diagnostic tools from Hogrefe, UTB, Ernst Reinhardt, Kohlhammer, and Liber, and is a partner at NeuroSupportSolutions International AB. CC is a full-time employee of Genentech and owns stocks or RSUs in Roche Holdings, Ltd. MA is the UK chief investigator for a trial sponsored by Roche (a phase II, randomised, double-blind, placebo-controlled, parallel group study to evaluate the safety, efficacy, and pharmacodynamics of 52 weeks of treatment with basmasanil in participants aged 2 to 14 years old with dup15q syndrome followed by a 2-year optional open-label extension). LB served on an advisory board to Kingdom therapeutics in 2022.

For peer review only - http://bmjopen.bmj.com/site/about/guidelines.xhtml

WORD COUNT: 3684

For peer review only

For peer review only - http://bmjopen.bmj.com/site/about/guidelines.xhtml

Location	Site	Role*
Stockholm, Sweden	Karolinska Institutet	R
London, UK	King's College London	R, L
	Evelina London, Guy's & St Thomas NHS Foundation Trust	R
Dublin, Ireland	Trinity College Dublin	R
Paris, France	Assistance Publique-Hopitaux de Paris	R
	Commissariat a l'Energie Atomique et aux Energies Alternatives	S
	Institut Pasteur	S
Heidelberg, Germany	Heidelberg University Hospital	R
Treviso, Italy	Associazione La Nostra Famiglia Istituto Scientifico Eugenio Medea	R
Pisa, Italy	Fondazione Stella Maris	R
Rome, Italy	Ospedale Pediatrico Bambino Gesù	R
Troina, Italy	Associazione Oasi Maria SS. Onlus	R
Barcelona, Spain	Hospital Clínic de Barcelona & Fundacio Clinic per a la Recerca Biomedica	R
	Fundació Sant Joan de Déu	R
Coimbra, Portugal	University of Coimbra	R

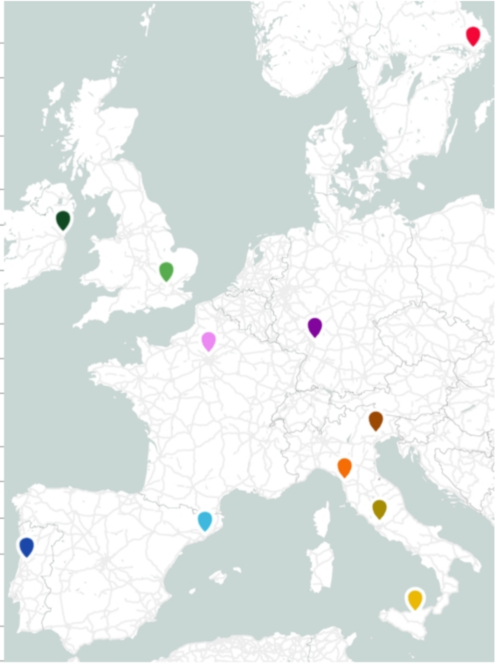

**R=Recruitment, L=Lead centre, S=Sample storage, sequencing, analysis*

Figure 1. EAGER collaborating centres

1500x1060mm (38 x 38 DPI)

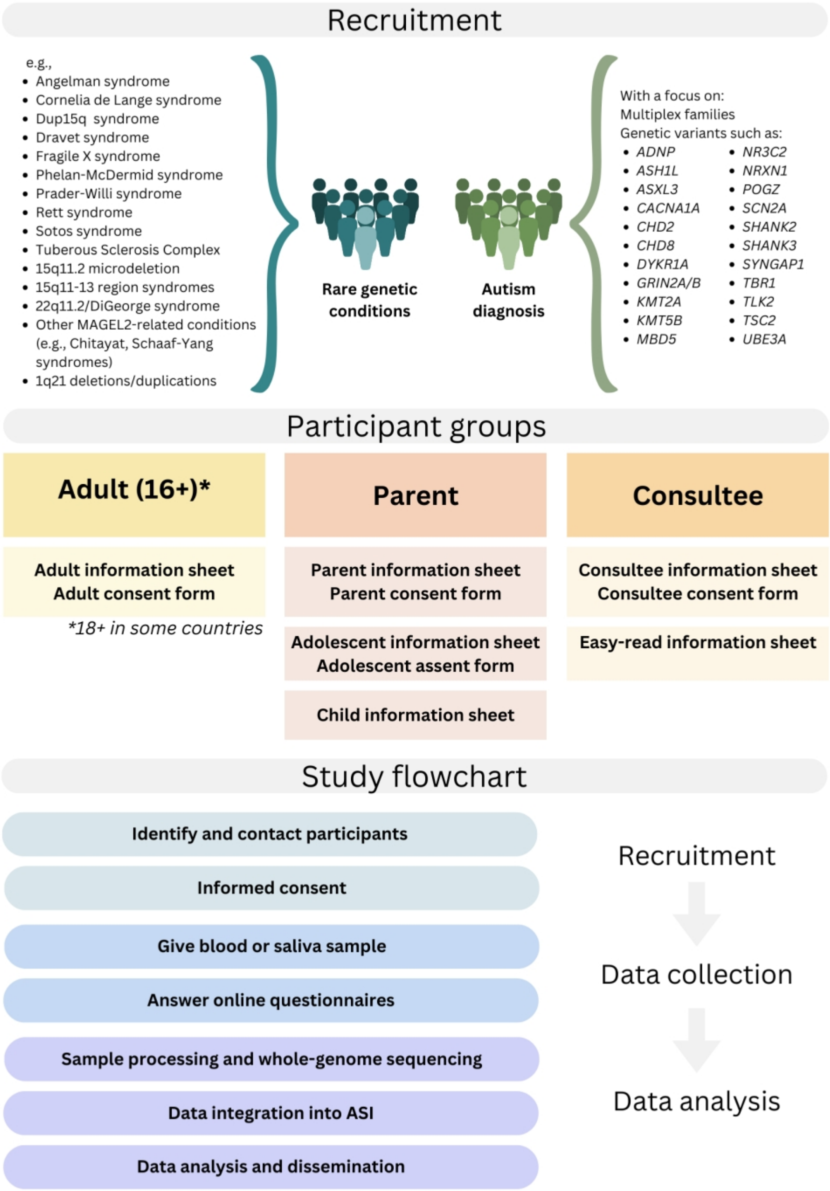

Figure 2. EAGER protocol summary

1060x1500mm (38 x 38 DPI)
